# Three‐Dimensional Vessels‐on‐a‐Chip Based on hiPSC‐derived Vascular Endothelial and Smooth Muscle Cells

**DOI:** 10.1002/cpz1.564

**Published:** 2022-10-17

**Authors:** Merve Bulut, Marc Vila Cuenca, Mees de Graaf, Francijna E. van den Hil, Christine L. Mummery, Valeria V. Orlova

**Affiliations:** ^1^ Department of Anatomy and Embryology Leiden University Medical Center Leiden The Netherlands; ^2^ Department of Applied Stem Cell Technologies University of Twente Enschede The Netherlands

**Keywords:** differentiation, endothelial cells, human induced pluripotent stem cells, neural crest, vascular smooth muscle cells, vessels‐on‐a‐chip

## Abstract

Blood vessels are composed of endothelial cells (ECs) that form the inner vessel wall and mural cells that cover the ECs to mediate their stabilization. Crosstalk between ECs and VSMCs while the ECs undergo microfluidic flow is vital for the function and integrity of blood vessels. Here, we describe a protocol to generate three‐dimensional (3D) engineered vessels‐on‐chip (VoCs) composed of vascular cells derived from human induced pluripotent stem cells (hiPSCs). We first describe protocols for robust differentiation of vascular smooth muscle cells (hiPSC‐VSMCs) from hiPSCs that are effective across multiple hiPSC lines. Second, we describe the fabrication of a simple microfluidic device consisting of a single collagen lumen that can act as a cell scaffold and support fluid flow using the viscous finger patterning (VFP) technique. After the channel is seeded sequentially with hiPSC‐derived ECs (hiPSC‐ECs) and hiPSC‐VSMCs, a stable EC barrier covered by VSMCs lines the collagen lumen. We demonstrate that this 3D VoC model can recapitulate physiological cell‐cell interaction and can be perfused under physiological shear stress using a microfluidic pump. The uniform geometry of the vessel lumens allows precise control of flow dynamics. We have thus developed a robust protocol to generate an entirely isogenic hiPSC‐derived 3D VoC model, which could be valuable for studying vessel barrier function and physiology in healthy or disease states. © 2022 The Authors. Current Protocols published by Wiley Periodicals LLC.

**Basic Protocol 1**: Differentiation of hiPSC‐VSMCs

**Support Protocol 1**: Characterization of hiPSC‐NCCs and hiPSC‐VSMCs

**Support Protocol 2**: Preparation of cryopreserved hiPSC‐VSMCs and hiPSC‐ECs for VoC culture

**Basic Protocol 2**: Generation of 3D VoC model composed of hiPSC‐ECs and hiPSC‐VSMCs

**Support Protocol 3**: Structural characterization of 3D VoC model

## INTRODUCTION

Blood vessels are lumenized tubes composed of endothelial cells (ECs), which form the inner vessel wall lining, and surrounding mural cells that cover the outer endothelium. Mural cells are subdivided into pericytes and vascular smooth muscle cells (VSMCs) depending on their location within the vascular bed in vivo. Crosstalk between ECs and mural cells is essential for proper function and integrity of blood vessels (Gaengel, Genové, Armulik, & Betsholtz, 2009) and ensures vessel stability. Aberrant interactions between ECs and mural cells have been implicated in many pathological states, including congenital vascular diseases, diabetic retinopathy, atherosclerosis, and coronary artery disease (Owens, Kumar, & Wamhoff, 2004; Sweeney & Foldes, 2018).

Microphysiological systems integrating human vascular cells, also referred as Vessel‐on‐Chip (VoC) devices, are considered good representations of human vasculature in vitro because microfluidic flow promotes the recapitulation of key physiological aspects of vascular biology. Although the majority of current VoC platforms incorporate primary human cells, because of the batch‐to‐batch and genetic variability of primary vascular cells, as well as their restricted availability, there is a growing interest in utilizing human induced pluripotent stem cell (hiPSC)‐derived vascular cells in VoC platforms (Cochrane et al., 2019). With the emergence of reproducible methods for directed differentiation, hiPSCs generated from heathy individuals and patients with various conditions have already demonstrated value as a renewable source of vascular cells for disease modeling and drug discovery (Passier, Orlova, & Mummery, 2016; Samuel, Duda, Fukumura, & Jain, 2015; Shi, Inoue, Wu, & Yamanaka, 2016). In this regard, we and others have generated ECs from hiPSC lines and described robust protocols for the purification and cryopreservation of hiPSC‐ECs, allowing the derivation and storage of large batches of cells from the same donor and with features similar to those of primary ECs (Orlova et al., 2014a, 2014b; Patsch et al., 2015; Rufaihah et al., 2013; Zhang et al., 2017). VSMCs are also phenotypically highly dependent on their cell or lineage of origin (Majesky, 2007). We and others have previously derived hiPSC‐VSMCs from the neural crest (NC), the primary source of VSMCs in the cerebral vasculature (Cheung, Bernardo, Trotter, Pedersen, & Sinha, 2012; Dash et al., 2016; Granata et al., 2016; Halaidych, Cochrane, van den Hil, Mummery, & Orlova, 2019a; Wanjare, Kuo, & Gerecht, 2013, 2014).

Here, we describe two detailed protocols for (1) differentiating VSMCs from hiPSCs (Basic Protocol 1) and (2) incorporating hiPSC‐derived ECs and VSMCs into 3D‐engineered VoCs (Basic Protocol 2); the method described is a refinement of one we have used previously (de Graaf et al., 2019; Vila Cuenca et al., 2021). Our method is a robust and reproducible approach to generating 3D multi‐cell‐type VoC models entirely based on hiPSC that enable functional assays integrating flow dynamics for vascular disease modeling. Prior to incorporation in the 3D VoC model, hiPSC‐derived neural crest cells (hiPSC‐NCCs) and VSMCs are differentiated and cryopreserved in large batches following Basic Protocol 1. The differentiation efficiency of hiPSC‐NCCs and VSMCs is assessed by following Support Protocol 1. hiPSC‐ECs are derived and cryopreserved following a previously published protocol (Orlova et al., 2014b). Cryopreserved vials of hiPSC‐ECs and VSMCs are thawed and cultured for 4 days (Support Protocol 2) before being integrated into the microfluidic devices. The devices are fabricated using the soft‐lithography technique, and collagen type I lumenized scaffolds are created using an optimized viscous finger patterning (VFP) method for subsequent cell seeding (Basic Protocol 2). The structure of the 3D vessel barrier thus generated has a consistent geometry over its entire length, and its quality can be assessed following Support Protocol 3.

## Basic Protocol 1 DIFFERENTIATION OF hiPSC‐VSMCs

**Figure 1:**
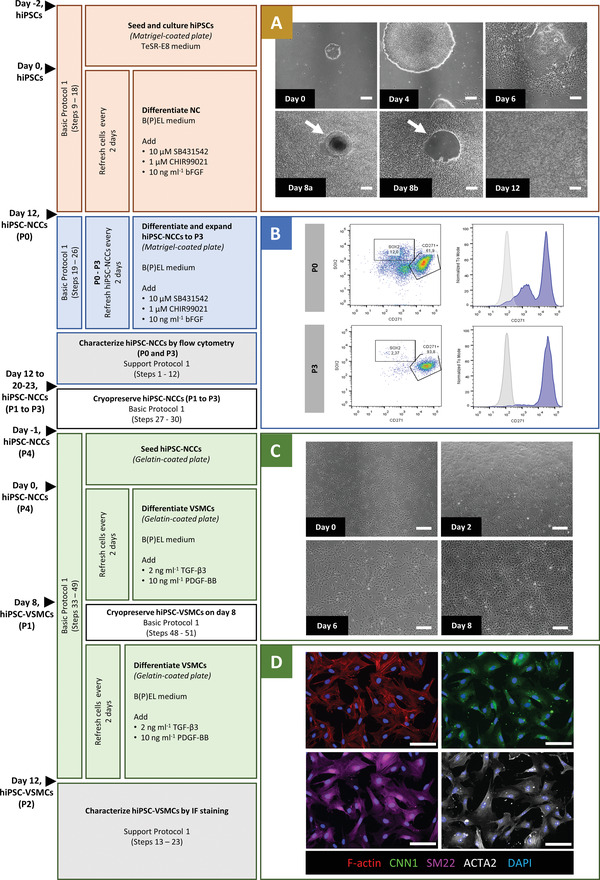
Timeline of Basic Protocol 1 and Support Protocol 1 with representative images at right. (**A**) Bright‐field images of cellular morphology for the NC differentiation procedure (days 0, 4, 6, 8, and 12). Colony centers on day 8 are indicated with a white arrow before (day 8a) and after (8b) elimination by scraping. (**B**) Characterization of hiPSC‐NCCs (P0 and P3) by flow cytometry analysis for an NC‐specific marker (CD271) and a pluripotency‐specific marker (SOX2). (**C**) Bright‐field images of initial cell density and cellular morphology for VSMC differentiation procedure (days 0, 2, 6, and 8). (**D**) Immunofluorescence images showing fully differentiated hiPSC‐VSMCs (day 12) (F‐actin; red, CNN1; green, SM22; magenta, ACTA2; grey, DAPI; blue). Scale bars: 200 μm in all bright‐field images, 100 μm in immunofluorescence images. hiPSCs, human induced pluripotent stem cells; NC, neural crest; NCC, neural crest cells; VSMC, vascular smooth muscle cell.

This protocol provides a method for efficient derivation of hiPSC‐VSMCs through NC intermediates. It has previously proven robust and reproducible across multiple independent hiPSC lines (Halaidych et al., 2019a; Vila Cuenca et al., 2021). VSMC differentiation from hiPSCs is divided into three steps (Fig. 1). The first step is seeding and culture of hiPSCs followed by NC induction for 12 days (Fig. 1A). The initial seeding density of hiPSCs is critical for the differentiation efficiency. NC formation is induced by combining the inhibitor of glycogen synthase kinase 3, CHIR99021, to activate canonical WNT signaling with basic fibroblast growth factor and activin/nodal inhibitor SB431542. On day 12 (passage 0, P0), the differentiation efficiency can be assessed by flow cytometry analysis for reduced expression of the pluripotency marker SOX2 and increased expression of the NC marker CD271 (Support Protocol 1). The second step is the further differentiation and expansion of the hiPSC‐NC cells (hiPSC‐NCCs) from P0 to P3 for 8‐11 days using the same (defined) medium used for differentiation. At P3, flow cytometry analysis should show a high total yield of CD271^+^ NC cells and high reproducibility across different lines and batches (Fig. 1B). At this stage, the cells can be cryopreserved and thawed for further differentiation to VSMCs. The third step is VSMC induction for 12 days (Fig. 1C). Both fresh and cryopreserved hiPSC‐NCCs (P3) can be used to differentiate VSMCs. The initial seeding density of hiPSC‐NCCs is important for a high differentiation yield. Differentiation to VSMCs is induced using PDGF‐BB and TGF‐β3 for 12 days. Ideally, hiPSC‐VSMCs are cryopreserved on day 8 of VSMC induction to make a large batch of cells from the same differentiation batch that can be used over long periods in multiple experiments. Cryopreserved vials can later be thawed for further differentiation until day 12 (Support Protocol 2) for use in functional assays and characterization (Support Protocol 1).

### Materials

Vitronectin‐coated 6‐well plate (see recipe)
TeSR™‐E8™ Kit for hESC/hiPSC (Stemcell Technologies, cat. no. 05990)
Human induced pluripotent stem cells (hiPSCs):
  LUMC0054 (LU54, generated from kidney epithelial cells isolated from cells in urine, http://hpscreg.eu/cell‐line/LUMCi001‐A; Halaidych et al., 2018)
  Allen Cell Collection line AICS‐0016 (ACTB‐mEGFP, generated from skin fibroblasts, https://hpscreg.eu/cell‐line/UCSFi001‐A‐3; Roberts et al., 2017)
Gentle Cell Dissociation Reagent (GCDR; Stemcell Technologies, cat. no. 07174)
Cell Adhere™ dilution buffer (Stemcell Technologies, cat. no. 07183)
Dulbecco's modified Eagle's medium/nutrient mixture F‐12 (DMEM/F12) medium (Gibco™; Thermo Fisher Scientific, cat. no. 31331028)
Matrigel‐coated 6‐well plate (see recipe)
B(P)EL medium (see recipe)
NC differentiation medium (see recipe)
Dulbecco's phosphate‐buffered saline (DPBS; Life Technologies, cat. no. 14190)
TrypLE™ Express Enzyme (1×), no phenol red; Gibco™, Thermo Fisher Scientific, cat. no. 12604021)
CryoStor® CS10 cryopreservation medium (Stemcell Technologies, cat. no. 07930)
Gelatin‐coated 6‐well plate (see recipe)
VSMC differentiation medium (see recipe)

6‐well suspension plate (Greiner Bio‐One, cat. no. 657185)
Tissue culture incubator, 37°C, 5% CO_2_
28‐cm cell scraper, blue (Greiner Bio‐One, cat. no. 541070)
15‐ml tubes (Greiner Bio‐One, cat. no. 188271)
Stereomicroscope (Leica MZ6 or equivalent)
6‐well culture plate (Greiner Bio‐One, cat. no. 657160)
Cryovials (Greiner Bio‐One, cat. no. 123263)
Mr. Frosty™ Freezing Container

### Passaging of hiPSCs for maintenance

Passage cells once a week when hiPSC colonies become relatively big and start to contact each other (∼90% confluency).

1 Prewarm a vitronectin‐coated 6‐well suspension plate, or freshly coat two wells of a 6‐well suspension plate with vitronectin (see recipe). Prewarm TeSR‐E8 to room temperature.
2 Before beginning cell dissociation, remove regions of spontaneous differentiation by scraping them off with a 200‐μl pipet tip. Aspirate TeSR‐E8 containing differentiated parts of the culture.
  Spontaneous differentiation can emerge in the edges of hiPSC colonies and can be identified by their heterogeneous morphology. Removing these regions is only necessary if it is >5% of the hiPSC colonies.
3 Add 1 ml GCDR to each well of a fresh 6‐well plate and incubate 3‐6 min at room temperature.
  The timing of GCDR dissociation varies depending on the hiPSC line/clone. Determine adequate incubation time by visual inspection. A prolonged dissociation time may lead to cell detachment.
4 Aspirate the GCDR and add 1 ml TeSR‐E8 to each plate well. Gently detach cell colonies by scraping them with a cell scraper.
5 Transfer cell aggregates into a 15‐ml tube using a 1000‐µl pipet tip. Pipet up and down gently once or twice with a 1000‐µl pipet tip to break up large aggregates.
  It is critical to break up large pieces of colonies to increase their adherence and survival after seeding.
6 Wash the vitronectin‐coated 6‐well plate (from step 1) with 1 ml Cell Adhere dilution buffer per well and then add 2 ml TeSR‐E8.
7 Add the small aggregates from step 5 to each well at a split ratio of 1:20‐1:40. Move the plate in a cross‐like pattern to distribute cells evenly, and place it the incubator at 37°C and 5% CO_2_.
  It is important to ensure that the cell suspension is well mixed in the tube. To do so, gently tap the bottom of the 15‐ml tube before taking up the cell suspension from step 5.
8 Refresh cells with 2 ml TeSR‐E8 once a day starting 24 hr after passaging.
  A larger amount of TeSR‐E8 can be added when the cells become relatively dense 5‐6 days after passaging.

### Passaging of hiPSCs for NC differentiation (day –2)

Passage hiPSCs when they reach 90% confluency, as with the passaging for maintenance above.

9 Prewarm a Matrigel‐coated 6‐well plate, or freshly coat two wells of a 6‐well plate (see recipe). Prewarm TeSR‐E8 medium to room temperature.
10 Dissociate hiPSC colonies as described in steps 3‐5.
11 Aspirate Matrigel and add 2 ml TeSR‐E8 to each well of 6‐well plate.
12 Add 20‐25 μl of cell suspension (step 10) to each well at a split ratio of 1:30‐1:40. Distribute cells evenly by moving the plate in a cross‐like pattern, and place it in the incubator at 37°C and 5% CO_2_.
  The seeding density of hiPSCs is important for their differentiation efficiency with low‐density cultures showing more differentiation. When hiPSCs are dissociated from a partially confluent well, a lower split ratio (1:20) is recommended.
13 Refresh each well with 2 ml TeSR‐E8 after 24 hr (day –1).

### Differentiation of hiPSC‐NCCs (from day 0 to 12)

*IMPORTANT NOTE*: Always prewarm medium to room temperature and add growth factors to the medium right before refreshing cells. Replace the cells in the 37°C, 5% CO_2_ incubator after refreshing.

14 NC induction: 48 hr after passaging (step 12), aspirate TeSR‐E8 medium and add 2 ml NC differentiation medium in each well of the 6‐well plate (day 0).
  Prior to NC induction, using 20‐30 colonies ranging from 200 to 400 µm in size on day 0 is expected to result in efficient differentiation.
15 Refresh each well with 2 ml NC differentiation medium every 2 days until day 12.
  At days 5‐7, differentiated NCCs will start to migrate from the colony edges as a monolayer.
16 Gently remove the cells in the centers of initial colonies by scraping them with a 200‐μl pipet tip (day 8 or 9). This can be done under a stereomicroscope.
  As more cells migrate, mechanical elimination of the cells in the center of the colonies improves the purification of a neural crest population. Representative images of a single colony center on day 8 are shown in Figure 1A pointing to the site of elimination (day 8a), as well as showing the “empty” colony center after elimination (day 8b).
17 After removing the cells in the center of the colonies, refresh each well with 2 ml NC differentiation medium (day 8 or 9). Refresh each well again 2 days after mechanical elimination (day 10 or 11).
18 Passage the cells with a split ratio of 1:2 when they reach 80% confluency (day 12) for further differentiation and expansion following steps 19‐25.
  The yield of differentiation is expected to be 2‐3 million hiPSC‐NCCs in total on day 12 (P0). In this step, the differentiation efficiency of hiPSC‐NCCs can be determined by flow cytometry (Support Protocol 1). More than 50% of hiPSC‐NCCs should be CD271^+^ on day 12 (P0); lower percentages will result in less efficient expansion during later steps.

### Differentiation and expansion of hiPSC‐NCCs P0–P3 (from day 12 to day 20 or 23)

19 Prewarm a Matrigel‐coated 6‐well plate, or freshly coat four wells of a 6‐well plate (see recipe). Prewarm B(P)EL medium to room temperature and freshly add the growth factors to prepare NC differentiation medium (see recipe).
20 Aspirate NC differentiation medium and add 2 ml DPBS to each well of a 6‐well plate.
21 Aspirate DPBS and add 0.5 ml TrypLE™ to each well. Incubate for 3 min at room temperature.
22 Add 1.5 ml of B(P)EL to each well to dilute TrypLE. Transfer cell suspension to a 15‐ml tube using a 1000‐µl pipet tip. Pipet up and down gently once or twice with a 1000‐µl pipet tip to break up the cell aggregates.
23 Centrifuge the tube 3 min at 300 × *g*, room temperature. Aspirate the supernatant.
24 Resuspend the cell pellet in freshly prepared 8 ml NC differentiation medium.
25 Aspirate Matrigel from four wells of 6‐well plate and add 2 ml hiPSC‐NCCs from step 24 to each well (for a split ratio of 1:2). Distribute the cells evenly by moving the plate in a cross‐like pattern, and place it in the incubator at 37°C and 5% CO_2_.
  The cell monolayer is expected to be confluent in 3‐4 days.
26 Follow the same steps for passaging to P2 and to P3.
  After the first passage, cells start to proliferate more rapidly; therefore, the split ratio of passaging should be modified. For P2, use a split ratio of 1:4 or 1:5, and for P3, a split ratio of 1:5 or 1:6.
  The yield of hiPSC‐NCCs at P3 is expected to be 40‐60 million cells total, with >90% being CD271^+^.
  Efficient induction of hiPSC‐NCC differentiation can also be assessed by immunofluorescence staining and gene expression analysis (Halaidych et al., 2019a). hiPSC‐NCCs can be passaged up to P7.

### Cryopreservation of hiPSC‐NCCs P1‐P3

*NOTE*: It is best to cryopreserve hiPSC‐NCCs at P1‐P3 in order to create batches from the same differentiation.

27 Prechill a Mr. Frosty^TM^ Freezing Container to 4°C. Label cryovials and place on ice.
28 Dissociate hiPSC‐NCCs as described in steps 20‐23.
29 Resuspend the cell pellet in cold CryoStor® CS10 cryopreservation medium (1 ml per 20 cm^2^ growth surface). Transfer 500 µl of cell suspension to each cryovial (10 cm^2^ per cryovial).
  hiPSC‐NCCs and CS10 cryopreservation medium should be kept on ice. Cryovials should be kept on ice during transferring the cells.
30 Place all cryovials into a Mr. Frosty™ Freezing Container and leave at −80°C for 24 hr. Transfer all cryovials into liquid nitrogen for prolonged storage.
  Differentiation at the scale described in this protocol should yield 80‐120 vials that can be cryopreserved at P3 (500,000 cells per vial).

### Differentiation of VSMCs from hiPSC‐NCCs in 12 days (days 0‐12)

NOTE: VSMC differentiation steps can be followed by using either a cryopreserved vial or fresh hiPSC‐NCCs at P3 from step 26.

31 On day –1, prewarm a 0.1% gelatin‐coated 6‐well plate, or freshly coat two wells of a 6‐well plate. Prewarm B(P)EL medium to room temperature and add the growth factors to prepare NC or VSMC differentiation medium.
32 If using cryopreserved hiPSC‐NCCs, remove cryovial (P3) from liquid nitrogen and thaw in a water bath at 37°C until only a small ice crystal remains. Transfer the cells into a 15‐ml tube containing 5 ml B(P)EL. Wash cryovial once with 1 ml B(P)EL to collect remaining cells.
  The thawing procedure should be performed as quickly as possible. Move the cryovial in the water bath in a circle to thaw. Pipet cells gently to reduce mechanical stress.
33 Gently homogenize the cell suspension with a 5‐ml pipet and take a small aliquot to use in counting cells. Centrifuge the 15‐ml tube 3 min at 300 × *ɡ*, room temperature. Aspirate supernatant.
34 Resuspend the cell pellet in NC differentiation medium to reach 1.25 × 10^5^ cells/ml.
35 Aspirate gelatin from two wells of a 6‐well plate and add 2 ml cell suspension to each well. Distribute the cells evenly by moving the plate in a cross‐like pattern, and place it in the incubator at 37°C and 5% CO_2_ (day –1).
36 At 24 hr after seeding, start VSMC differentiation. Refresh each well with 2 ml VSMC differentiation medium (day 0).
  24 hr after seeding (day 0), hiPSC‐NCCs should have a density approximating that shown in Figure 1C. This is the optimal density to start VSMC differentiation.
37 On day 2, refresh each well with 2 ml VSMC differentiation medium.
38 Passage the cells at a split ratio of 1:4 (day 4). Prewarm 0.1% gelatin‐coated 6‐well suspension plates, or freshly coat eight wells.
39 Aspirate VSMC differentiation medium and add 2 ml DPBS to each well of a 6‐well plate.
40 Aspirate DPBS and add 0.5 ml TrypLE™ to each well. Incubate 3 min at room temperature.
41 Add 1.5 ml B(P)EL to each well to dilute TrypLE™. Transfer cell suspension to a 15‐ml tube using a 1000‐µl pipet tip. Pipet up and down gently once or twice with a 1000‐µl pipet tip to dissociate the large aggregates.
42 Centrifuge the tube 3 min at 300 × *g*, room temperature. Aspirate the supernatant.
43 Resuspend the cell pellet in 16 ml VSMC differentiation medium.
44 Aspirate gelatin from eight wells of 6‐well suspension plates and add 2 ml cell suspension to each well (at a split ratio of 1:4). Distribute the cells evenly by moving the plates in a cross‐like pattern, and place them in the incubator at 37°C and 5% CO_2_.
45 On day 6, refresh each well with 2 ml VSMC differentiation medium.
  It is best to cryopreserve VSMCs on day 8 to make a batch of cells from the same differentiation for multiple experiments. Cryovials can be thawed and further differentiated in VSMC differentiation medium until day 12 (Support Protocol 2, steps 1‐6).
46 Alternatively, on day 8, passage VSMCs as described in steps 39‐44.
47 On day 10, refresh each well with 2 ml VSMC differentiation medium.
  On day 12, hiPSC‐VSMCs are expected to be fully differentiated and ready for use in functional assays.

### Cryopreservation of VSMCs (day 8)

48 Prechill a Mr. Frosty^TM^ Freezing Container to 4°C. Label cryovials from each differentiation and place on ice.
49 Dissociate hiPSC‐VSMCs as described in steps 39‐42.
50 Resuspend the cell pellet in cold CryoStor® CS10 cryopreservation medium to get a final concentration of 20 cm^2^/ml. Transfer 500 µl of cell suspension to each cryovial (10 cm^2^ per cryovial).
  hiPSC‐VSMCs and CS10 cryopreservation medium should be kept on ice. Cryovials should be kept on ice during transfer of the cells.
51 Place all cryovials in a Mr. Frosty™ Freezing Container and leave at −80°C for 24 hr. Then transfer all cryovials into liquid nitrogen for prolonged storage.

## Support Protocol 1 CHARACTERIZATION OF hiPSC‐NCCs and hiPSC‐VSMCs

This protocol describes characterization of hiPSC‐NCCs and hiPSC‐VSMCs. On day 12 of NC induction (P0), the efficiency of differentiation can be determined through flow cytometry by assessing the expression of pluripotency markers (e.g., SOX2) and NC markers (e.g., CD271). More than 50% of the cells should be CD271^+^ NCCs. The analysis should be repeated at P3 (day 20 or 23), when the total CD271^+^ yield is expected to be higher (>90%) and the expression of SOX2 lower than at P0 (Fig. 1B). hiPSC‐NCCs at P3 can be cryopreserved and thawed for VSMC induction. On day 12 of VSMC induction, hiPSC‐VSMCs are fully differentiated and ready for functional characterization. At this stage, hiPSC‐VSMCs exhibit increased expression of contractile proteins calponin 1 (CNN1), α‐smooth muscle actin (αSMA; ACTA2), and SM22 (TAGLN), which can be assessed by immunofluorescence staining (Fig. 1D).

### Materials

P3 hiPSC‐NCCs (Basic Protocol 1, step 24)
Dulbecco's phosphate‐buffered saline (DPBS; Life Technologies, cat. no. 14190)
TrypLE™ Express Enzyme (1×), no phenol red (Gibco™; Thermo Fisher Scientific, cat. no. 12604021)
Fluorescence‐activated cell sorting buffer (FACSB; see recipe)
BD Cytofix/Cytoperm kit (BD Biosciences, cat. no. 554714)
Fluorescent‐conjugated antibodies:
  Anti‐human CD271, BV421 conjugated (used at 1:100 dilution; BD Biosciences, cat. no. 562562)
  Anti‐human SOX2, A488 conjugated (used at 1:50 dilution; eBiosciences, cat. no. 53‐9811‐80)
Day 12 hiPSC‐VSMCs (Basic Protocol 1, step 47, or Support Protocol 2, step 6)
B(P)EL (see recipe)
4% (w/v) PFA (see recipe)
0.1% (v/v) Triton X‐100 (see recipe)
1% (w/v) BSA in DPBS (see recipe)
Primary antibodies:
  Anti‐human Calponin, Mouse (used at 1:200 dilution; Sigma‐Aldrich, cat. no. C2687)
  Anti‐human Smooth Muscle Actin 1A4, Mouse (used at 1:200 dilution; Sigma‐Aldrich, cat. no. A2547)
  Anti‐human SM22 (TAGLIN/Transgelin), Rabbit (used at 1:200 dilution; Abcam, cat. no. AB14106)

100‐μm sterile filters (CellTrics, cat. no. 04‐004‐2328)
5‐ml round‐bottom FACS tube (BD Biosciences, cat. no. 352058)
MACSQuant® VYB Flow Cytometer (Miltenyi Biotec, cat. no. 130‐096‐116)
Tissue culture incubator, 37°C, 5% CO_2_
96‐well plate
Falcon® 96‐well Black/Clear Imaging Microplate (Corning Life Sciences, cat. no. 353219)
Sealing film, 10 cm × 38 m (Parafilm M, cat. no. 291‐1213)
EVOS M7000 imaging system (Thermo Fisher Scientific, cat. no. AMF7000)

### Characterization of hiPSC‐NCCs by flow cytometry (P0 and P3)

1 Aspirate NC differentiation medium from hiPSC‐NCCs (P0 or P3) in 6‐well plates and add 2 ml DPBS to each well.
2 Aspirate DPBS and add 0.5 ml TrypLE™ to each well. Incubate 3 min at room temperature.
3 Add 1 ml FACSB to each well to stop the dissociation, and wash the cells by pipetting up and down once or twice.
4 Place a 100‐μm CellTrics sterile filter on top of a 5‐ml FACS tube. Transfer an aliquot of hiPSC‐NCC suspension into a 5‐ml FACS tube for analysis. Centrifuge the tube 3 min at 300 × *g*, room temperature.
5 Wash the cell suspension with 1 ml FACSB and centrifuge the tube 3 min at 300 × *g*, room temperature. Aspirate the supernatant and leave around 50 µl FACSB with the cell pellet.
6 Add fluorescent‐conjugated FACS antibodies to the cell suspension to the desired working concentration in FACSB. Resuspend cells by flicking the bottom of the tube. Incubate in the dark for 60 min at 4°C.
  CD271 antibody is used for the characterization of NCCs. Details of the antibodies are given in the Materials list.
7 Turn off the light of the cell culture hood. Wash the cell suspension with 1 ml FACSB and centrifuge the tube 3 min at 300 × *g*, room temperature.
  For intracellular staining, fixation and permeabilization steps are required, e.g., using the BD Cytofix/Cytoperm™ Fixation/Permeabilization Kit.
8 Aspirate the supernatant but leave ∼50 μl in the tube. Add 250 μl Fixation/Permeabilization solution (from BD Cytofix/Cytoperm kit). Resuspend the cells by flicking the bottom of the tube. Incubate in the dark for 20 min at 4°C.
9 Wash the cells once with 1 ml 1× BD Perm/Wash™ buffer (from kit) and centrifuge the tube 3 min at 300 × *g*, room temperature. Aspirate the supernatant but leave ∼50 μl in the tube.
10 Add fluorescent‐conjugated FACS antibodies to the cell suspension to the desired working concentration in 1× BD Perm/Wash™ buffer. Resuspend the cells by flicking the bottom of the tube. Incubate in the dark for 20 min at 4°C.
  Intracellular staining with SOX2 antibody is used to determine whether residual undifferentiated hiPSCs are present. Details of the antibodies are listed in the Materials section.
11 Wash the cells once with 1 ml 1× BD Perm/Wash™ buffer and centrifuge the tube for 3 min at 300 × *g*, room temperature. Resuspend the cells in 1 ml 1× BD Perm/Wash™ buffer.
12 Analyze the samples by flow cytometry immediately or the next day.
  We used the MACSQuant VYB flow cytometer with the following instrument settings: Blue/488 FITC, A488: 525/50; yellow/561 PE: 586/15; APC: 661/20; APC‐Cy7: 750LP. FlowJo software was used for data analysis.
  The MACSQuant VYB laser setup may require compensation between PE and APC channels. Extra sample tubes are required for a compensation run before the analysis. Checking the setup and compensation procedure provided in the manual of the flow cytometer is recommended.
  Sample data of Support Protocol 1 are shown in Figure 1B.

### Characterization of hiPSC‐VSMCs using immunofluorescence staining

13 Freshly coat the required number of wells of a 96‐well Black/Clear Imaging Microplate with fibronectin (see recipe). Prewarm B(P)EL medium to room temperature.
14 Dissociate hiPSC‐VSMCs (day 12) as described in Basic Protocol 1, steps 39‐42. Resuspend the cell pellet in B(P)EL to 7.5 × 10^4^ cells per ml.
15 Aspirate fibronectin and add 200 µl cell suspension to each well. Distribute the cells evenly by moving the plate in a cross‐like pattern and place in the incubator at 37°C and 5% CO_2_.
16 After 24 hr, aspirate B(P)EL and add 200 µl of 4% (w/v) PFA to each well to fix the cells. Incubate for 10 min at room temperature.
17 Wash the cells three times with DPBS, 5 min per wash, at room temperature.
  The fixed cells can be stored in DPBS for up to 2 weeks at 4˚C.
18 Add 200 µl 0.1% (v/v) Triton‐X 100 in DPBS to each well to permeabilize the cells. Incubate 5 min at room temperature.
19 Add 200 µl 1% (w/v) BSA in DPBS to each well to block nonspecific sites. Incubate 1 hr at room temperature.
20 Dilute the primary antibodies in 1% (w/v) BSA in DPBS and add it to the corresponding wells. Incubate overnight at 4°C.
  Primary antibodies used for characterization of hiPSC‐VSMCs include those for ACTA2, CNN1, and SM22. Details of the antibodies are given in the Materials list.
21 Wash the cells three times with DPBS for 10 min per wash at room temperature.
22 Dilute the secondary antibodies in 1% (w/v) BSA in DPBS and add to the corresponding wells. Incubate 2 hr at room temperature.
  The selection of secondary antibodies can vary between users, and the dilutions should be empirically derived depending on the specific antibody, incubation time and temperature.
23 Wash the cells three times with DPBS for 15 min per wash at room temperature. Seal the plates with Parafilm and store in the dark at 4°C until microscopic imaging.
  The plates can be stored for prolonged periods in the cold and dark, although the highest quality images are obtained within 1‐2 weeks.

## Support Protocol 2 PREPARATION OF CRYOPRESERVED hiPSC‐VSMCs AND hiPSC‐ECs for VoC CULTURE

**Figure 2:**
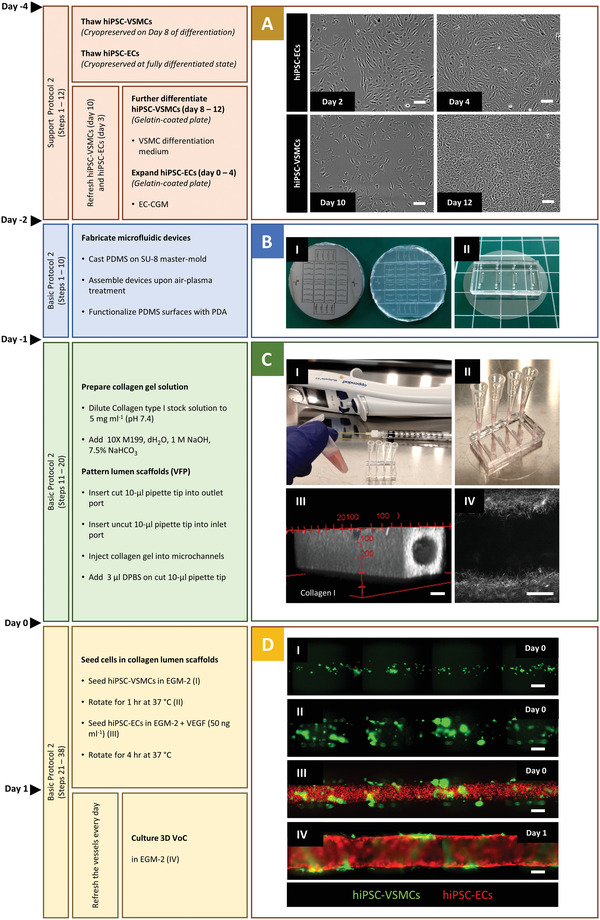
Timeline of Basic Protocol 2 and Support Protocol 2 with representative images at right. (**A**) Bright‐field images of cellular morphology and culture of thawed hiPSC‐ECs (days 2 and 4) and hiPSC‐VSMCs (days 10 and 12). (**B**) Images of microfluidic device fabrication procedure showing the SU‐8 mold, cured PDMS (I), and assembled microfluidic device (II). (**C**) Final collagen I solution (pH ∼7.4; pink) in a tube (I) together with the patterned devices (II). Representative 2P‐SHG image of collagen I showing the patterned 3D lumen scaffold (III) and collagen fibers inside the lumen (IV). (**D**) Representative immunofluorescence images showing the cell seeding procedure (day 0) for hiPSC‐VSMCs (I and II) and hiPSC‐ECs (III). The resulting 3D‐engineered vessel is shown on day 1 (IV). hiPSC‐VSMCs (ACTB; green) and hiPSC‐ECs (mCherry; red). Scale bars: 100 μm in all bright‐field images and 2P‐SHG images, 200 μm in immunofluorescence images of cell seeding procedure. hiPSCs, human induced pluripotent stem cells; ECs, endothelial cells; VSMCs, vascular smooth muscle cells; PDMS, polydimethylsiloxane; 2P‐SHG, 2‐photon second harmonic generation.

Vascular cells can be derived from hiPSCs using the protocols above and cryopreserved for further functional experiments. It is important to generate large batches of cells from the same differentiation for use in multiple VoC experiments. In this protocol, we describe how to prepare hiPSC vascular cells prior to VoC culture and functional assays. We previously described the cryopreservation of hiPSC‐VSMCs on day 8 of differentiation. Cryopreserved hiPSC‐VSMCs are thawed and cultured in VSMC differentiation medium for 4 days to reach the fully differentiated state (Fig. 2A). Additionally, hiPSC‐ECs are produced efficiently and cryopreserved in their fully differentiated state in large batches using the robust protocol we reported previously (Orlova et al., 2014b). hiPSC‐ECs are thawed and expanded in Endothelial Cell Complete Growth Medium (EC‐CGM) for 4 days. Both cell types reach >90% confluency after 4 days and are then ready to be integrated in the collagen lumenized scaffolds created following Basic Protocol 2.

### Materials

Gelatin‐coated 6‐well plate (see recipe)
Cryopreserved hiPSC‐derived VSMCs on day 8 (Basic Protocol 1, step 51)
B(P)EL (see recipe)
VSMC differentiation medium (see recipe)
Cryopreserved hiPSC‐derived ECs (Orlova et al., 2014b)
Endothelial Cell Complete Growth Medium (EC‐CGM; see recipe)

6‐well culture plate (Greiner Bio‐One, cat. no. 657160)
15‐ml tubes (Greiner Bio‐One, cat. no. 188271)
Tissue culture incubator, 37°C, 5% CO_2_

### Further differentiation of hiPSC‐VSMCs in 4 days (from day 8 to 12)

1 Prewarm a 0.1% gelatin‐coated 6‐well plate, or freshly coat four wells of a 6‐well plate. Prewarm B(P)EL to room temperature, and freshly add the growth factors to prepare VSMC differentiation medium.
2 Thaw the cryopreserved vial from day 8 of differentiation in a water bath at 37°C by swirling the cryovial until only a small ice crystal remains.
3 Transfer all cell suspension in the cryovial to a 15‐ml tube containing 5 ml B(P)EL. Centrifuge 3 min at 300 × *g*, room temperature. Aspirate the supernatant.
4 Resuspend the cell pellet in 8 ml VSMC differentiation medium.
5 Aspirate 0.1% gelatin from four wells of the 6‐well plate and add 2 ml cell suspension to each well (with a split ratio of 1:4). Distribute the cells evenly by moving the plate in a cross‐like pattern and place the plates in the incubator at 37°C and 5% CO_2_ (day 8).
6 Refresh each well with 2 ml VSMC differentiation medium (day 10).
  On day 12 of differentiation, hiPSC‐VSMCs are expected to become fully differentiated and should be >90% confluent with a yield of 700,000‐800,000 cells per well.

### Expansion of hiPSC‐ECs in 4 days (from day 0 to 4)

7 Prewarm a 0.1% gelatin‐coated 6‐well plate, or freshly coat six wells of a 6‐well plate (see recipe). Prewarm EC‐SFM and EC‐CGM to room temperature.
8 Thaw the vial of cryopreserved cells in a water bath at 37°C by swirling the cryovial until only a small ice crystal remains.
9 Transfer all cell suspension in the cryovial into a 15‐ml tube containing 8 ml EC‐SFM. Centrifuge 3 min at 300 × *g*, room temperature. Aspirate supernatant.
10 Resuspend the cell pellet in 12 ml EC‐CGM.
11 Aspirate 0.1% gelatin and add 2 ml cell suspension to each well (with a split ratio of 1:6). Distribute the cells evenly by moving the plate in a cross‐like pattern and place the plates in the incubator at 37°C and 5% CO_2_ (day 0).
12 Refresh the cells with EC‐CGM (day 3).
  On day 4 of expansion, hiPSC‐ECs should be >90% confluent with a yield of 250,000‐300,000 cells per well.

## Basic Protocol 2 GENERATION OF 3D VoC MODEL COMPOSED OF hiPSC‐ECs AND hiPSC‐VSMCs

**Figure 3:**
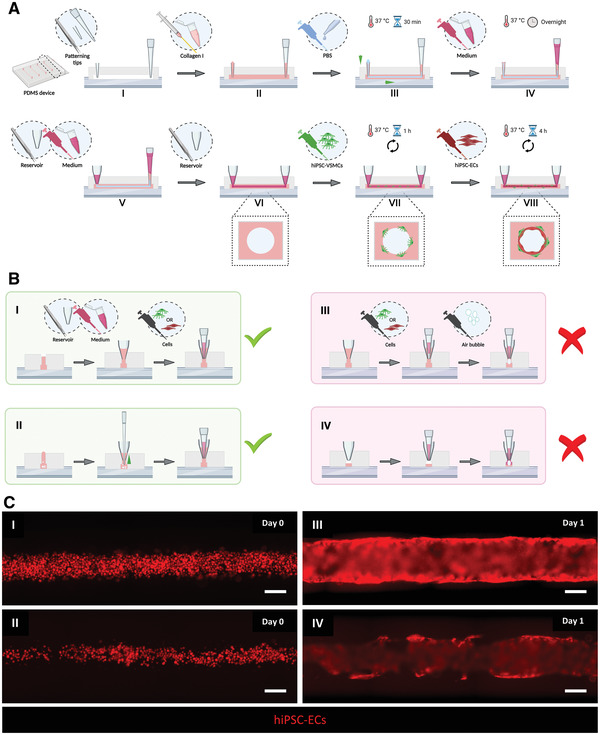
Schematic representation of collagen I patterning and cell seeding procedure. (**A**) VFP (I‐IV) and cell seeding (V‐VIII) protocols. (**B**) Left, correct handling practice for injecting cells via medium reservoirs (I) and for removing air bubbles from ports using gel‐load filter tips (II). Right, examples of poor handling practice, showing the potential ways that air bubbles may be injected (III and IV). (**C**) Representative fluorescence images showing initial hiPSC‐EC seeding density (day 0) for optimal density (I) and low seeding density (II) and the resulting hiPSC‐EC barrier (day 1) for optimal density (III) and low density (IV). hiPSC‐ECs (mCherry; red). Scale bars, 200 μm. VFP, viscous finger patterning; hiPSCs, human induced pluripotent stem cells; ECs, endothelial cells; VSMCs, vascular smooth muscle cells.

**Figure 4:**
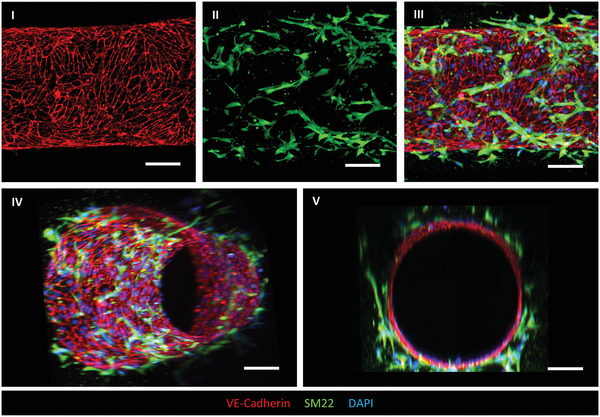
Structural characterization of 3D VoC model (Support Protocol 3). Representative confocal images of 3D vessels (day 3) showing hiPSC‐EC barrier (VE‐cadherin; red) and surrounding hiPSC‐VSMCs (SM22; green). A maximum projection of the halved *z*‐stack was made to visualize the bottom layer of the 3D vessel (I, II, and III). 3D reconstruction of full *z*‐stack shows the close interactions of hiPSC‐ECs (VE‐cadherin; red) and hiPSC‐VSMCs (SM22; green) (IV and V). Scale bar, 100 μm. hiPSCs, human induced pluripotent stem cells; ECs, endothelial cells; VSMC, vascular smooth muscle cell.

This protocol describes the generation of 3D‐engineered vessels composed of hiPSC‐ECs and hiPSC‐VSMCs inside a PDMS microfluidic chip. The protocol is divided into three steps (Fig. 2). The first step is fabricating the devices by casting polydimethylsiloxane (PDMS) silicone elastomer on a customized SU‐8 master mold using soft lithography. The master mold serves as a template to generate four straight microfluidic channels with fixed dimensions 0.5 mm (w) × 0.5 mm (h) × 10 mm (l) (Fig. 2B and 2I). After curing, PDMS is carefully peeled off, single devices are cut out, and the inlet/outlet ports of the microchannels are punched using biopsy puncher (1.2 mm) (Fig. 2B, II). To achieve watertight microfluidic channels, single devices are contact bonded with PDMS‐coated coverslips and then treated with air plasma. Subsequently, the PDMS surface is functionalized by polydopamine (PDA) to promote the binding of collagen type I. The second step is patterning the lumens inside the collagen type I in the microfluidic channels using viscous finger patterning (VFP; de Graaf et al., 2019), which is illustrated in Figure 3A. The success rate of the second step is >95% if the details outlined in the Critical Parameters are applied exactly. If necessary, 3D collagen scaffolds can be imaged at this point and analyzed using two‐photon second‐harmonic generation (2P‐SHG; Fig. 2C, III, and IV). The last step is the sequential seeding of hiPSC‐derived vascular cells into the collagen I lumen scaffolds, illustrated in Figure 3A. hiPSC‐VSMCs and hiPSC‐ECs are prepared as described in Support Protocol 2 (Fig. 2A) and subsequently seeded by injecting the cells into the lumen via the inlet port (Fig. 2D, I‐III). After each seeding step, the devices are slowly rotated to promote even coverage of the lumen scaffold. On day 1, a confluent hiPSC‐EC monolayer is formed that is surrounded by abluminal hiPSC‐VSMCs (Fig. 2D, IV). Functional assays can be performed on day 2 or 3 after cell seeding. Continuous and controlled perfusion can be done upon connection to the microfluidic pumps. Structural analysis in 3D is performed following Support Protocol 3, which reveals cell‐cell interactions in 3D (Fig. 4). More details about expected results are described in the Understanding Results section.

### Materials

Polydimethylsiloxane (PDMS) SYLGARD™ 184 Silicone Elastomer Kit (Dow Corning, cat. no. 101697)
2 mg/ml (w/v) poly(dopamine) (PDA; see recipe)
Distilled water (dH_2_O; Gibco™; Thermo Fisher Scientific, cat. no. 15230089)
5 mg/ml collagen type I, pH ∼7.4 (see recipe)
Dulbecco's phosphate‐buffered saline (DPBS; Life Technologies, cat. no. 14190)
Endothelial Cell Growth Medium 2 Kit (EGM‐2; Promocell, cat. no. C‐22111; see recipe)
Day 12 hiPSC‐VSMCs (Support Protocol 2, step 6), derived from Allen Cell Collection hiPSC line AICS‐0016 (ACTB‐mEGFP, generated from skin fibroblasts, https://hpscreg.eu/cell‐line/UCSFi001‐A‐3)
Day 4 hiPSC‐ECs (Support Protocol 2, step 12), derived from NCRM1 hiPSC line (NIH Center for Regenerative Medicine hiPSC line, https://hpscreg.eu/cell‐line/CRMi003‐A) engineered to express mCherry fluorescent protein under the control of the human cytomegalovirus (hCMV) early enhancer/chicken β‐actin (CAG) promoter (de Graaf et al., 2019)
TrypLE Express Enzyme (1×), no phenol red (Gibco™; Thermo Fisher Scientific, cat. no. 12604021)
Human VEGF (165) IS, premium grade (Miltenyi Biotec, cat. no. 130‐109‐386)

Vacuum desiccator (Fisher Scientific, cat. no. 467‐0090)
Custom‐made SU‐8 master‐mold
Oven (80°C)
Round cover glass, ø 35 mm #1 (Thermo Fisher Scientific, 11887902)
Laurell WS‐650HZ‐23NPP/UD2 spin coater
Razor blades (GEM Scientific, cat. no. 233‐1094)
Harris Uni‐Core punch Ø: 1.2 mm plus cutting mat (Merck, cat. no. WHAWB100028)
3M Scotch Magic Tape 810 (Lyreco, 3339099)
CUTE Vacuum Plasma System 220‐240V (Femto Science, serial no. FT20‐045, cat. no. CUTE‐1MPR)
Petri dish (100 mm; Greiner Bio‐One, cat. no. 664160)
Pressured nitrogen‐gas flow
CoolCLAVE Plus Ozone and UV Personal Sterilizer, 220V (Amsbio, E330220)
Laminar‐flow hood
Tissue culture incubator, 37°C, 5% CO_2_
P10 pipet tips (Greiner Bio‐One, cat. no. 741015)
Cut 10‐μl pipet tips (7 mm; see recipe)
1.5‐ml reaction tube (Greiner Bio‐One, cat. no. 616201)
Luer‐Lok™ 1‐ml syringe (BD, cat. no. 309628), prechilled on ice
Plastic needle, 20 AWG × 1.5 in., yellow (OK International Ltd., cat. no. TS20P‐1‐1/2PK), prechilled on ice
Electronic multi‐dispenser pipet (Multipette® E3; Eppendorf, cat. no. 4987000010)
Combitips® advanced, Biopur®, 0.2 ml (Eppendorf, cat. no. 0030089626)
Forceps, blunt tip
P1000 pipet tip (Greiner Bio‐One, cat. no. 740295)
Medium reservoirs for microchannels (cut 1000‐μl pipet tip; see recipe)
20‐µl Gel‐Load filter pipet tips (Greiner Bio‐One, cat. no. 775288)
Grant Instruments™ PTR‐35 rotator (Fisher Scientific, cat. no. 11481068)

### Fabrication of PDMS devices (day –2 or earlier)

1 Mix PDMS elastomer base and curing agent thoroughly in a 10:1 ratio and degas in a vacuum desiccator at room temperature.
2 Pour uncured PDMS mixture onto the SU‐8 master mold to a thickness of ∼5 mm.
3 Degas the PDMS until air bubbles are completely removed. Cure overnight in oven at 80°C.
4 Coat the cover glasses with a thin layer of PDMS using a spin‐coater (5 s at 1000 rpm, 30 s at 4000 rpm). Cure for at least 3 hr at 80°C.
5 Cool the mold to room temperature. Remove PDMS carefully in a direction parallel to the microchannels.
6 Cut out the individual chips using razor blades. Punch inlet and outlet ports at the opposite ends of the microchannels using a 1.2‐mm biopsy punch. Clean the PDMS surface with 3M Scotch® tape and store dust‐free.
  Dust particles should be cleaned before contact bonding of PDMS.
7 Place PDMS device (channel side up) and spin‐coated cover glass (coated side up) in a vacuum plasma system. Treat the surfaces with air‐plasma for 45 s at 50 W, 50 kHz.
8 Immediately bond the air‐plasma‐treated surfaces by pressing gently and evenly with fingers. Avoid breaking the cover glass.
  Optionally, the devices can be placed in the 80°C oven shortly to enhance the bonding. However, heat accelerates the hydrophobic recovery of the PDMS. The equipment and settings indicated in this protocol are sufficient to create a permanent contact bonding; enhancement by heating is not necessary.
  Assembled devices can be stored dry and dust‐free for up to 3 months at room temperature.

### Surface functionalization of the microfluidic channels to promote attachment of collagen I (day –2 or earlier)

9 Inject 10 µl PDA solution into the microchannels. Incubate 1 hr at room temperature. Wash the microchannels thoroughly with dH_2_O in a Petri dish. Dry completely using pressured nitrogen‐gas flow.
  The PDA solution darkens in color over time. A lack of color change might indicate ineffective reaction. If incubated for too long, PDA can hinder the optical transparency of PDMS.
  Partially wet channels will fail to efficiently attach to the collagen gel.
10 Sterilize the devices in CoolClave Ozone+UV chamber using the “UV” setting (30 min). The devices can be stored dry and in the dark up to 1 week at room temperature.

### Patterning of collagen I lumens using VFP technique (day –1)

*NOTE*: Follow aseptic techniques under sterile laminar hood. Use sterilized microfluidic devices and pipet tips.

11 Place 5 ml EGM‐2 medium in a slightly opened 15‐ml tube in the incubator at 37°C with 5% CO_2_ for at least 2 hr.
  Equilibrating the medium and devices inside the incubator prevents air bubbles emerging due to temperature differences.
12 Insert a cut 10‐μl pipet tip (7 mm) into the outlet port and an uncut 10‐μl pipet tip into the inlet port of the microchannel. Press the tips lightly until they reach the coverslip.
13 Prepare 5 mg/ml collagen I solution, pH ∼7.4 (see recipe ).
  Mix thoroughly until a homogeneous pink color is attained, indicating a near‐neutral pH of ∼7.4 (Fig. 2C and 2I). The color of the solution is the indication of pH, as 10× M199, a solution component, is supplemented with phenol red. Care should be taken in this step, as the pH defines mechanical property of collagen fibers and is crucial for cell viability. This procedure is reproducible given that adequate quantities of the reagents were used and were well mixed. Final solutions in the alkaline range, pH > 7.5 (purple in color), should be discarded. If the final solution is in acidic range, pH < 7.4 (yellow in color), pH can be adjusted to ∼7.4 (pink in color) by slowly adding droplet(s) of 1 M NaOH.
  If using collagen I stock solution from a different manufacturer, follow the instructions provided by the manufacturer.
14 Spin down the collagen solution for 10 s to remove air bubbles. Immediately place the solution back on ice. Take up the collagen solution in a prechilled 1‐ml syringe with a plastic needle.
15 Immediately inject the collagen solution steadily into the microchannels via the uncut 10‐μl pipet tip (inlet) until a meniscus forms on the cut 10‐μl pipet tip (outlet). Add a 3‐µl droplet of DPBS on top of the collagen I meniscus (outlet). Place the devices in the incubator at 37°C and 5% CO_2_ for 30 min to allow collagen polymerization.
  This step should be performed as quickly as possible. Limit the number of microchannels to pattern in each round and discard the collagen gel after 5 min. The yield of patterning drops dramatically when the same collagen gel is used for a long time.
  We recommend using a multi‐dispenser pipet to pattern several lumens at the same time. This way 24 microchannels can be patterned within 5 min. Alternatively, manual pipetting can be used and 12 channels can be patterned within 5 min.
16 Add 50 µl equilibrated EGM‐2 on top of the polymerized collagen via the uncut 10‐μl pipet tip (inlet). Place the devices in the incubator at 37°C with 5% CO_2_.
17 The next day, carefully remove the cut 10‐μl pipet tip from the outlet port in a smooth twisting motion using blunt‐tip forceps.
  It is critical to remove the tips gently in a twisting motion to prevent the deformation of the patterned lumens.
18 Insert a medium reservoir (cut 1000‐μl pipet tip) into the outlet port, and add 20 µl of equilibrated EGM‐2 to the medium reservoir using gel‐load filter tips.
  Check the ports of microchannels for air bubbles, which might emerge after removal of cut 10‐μl pipet tips. Remove the air bubbles with gel‐load filter tips as illustrated in Figure 3B, II.
19 Remove the uncut 10‐μl pipet tip from the inlet port in a smooth twisting motion using blunt‐tip forceps.
20 Insert a medium reservoir into the inlet port and proceed to cell seeding.
  It is advisable to use the patterned lumens for cell seeding the next day. Alternatively, the patterned lumens can be kept inside the incubator for up to 3 days before cell seeding.

### Seeding cells in 3D lumen scaffolds (day 0)

In the following steps, the confluent monolayers of hiPSC‐VSMCs (from Support Protocol 2; step 6) and hiPSC‐ECs (from Support Protocol 2; step 12) are dissociated sequentially.

21 Prewarm B(P)EL and EC‐SFM to room temperature. Place 5 ml EGM‐2 in a slightly opened 15‐ml tube in the incubator at 37°C with 5% CO_2_ and incubate at least 2 hr.
22 Dissociate the monolayer of hiPSC‐VSMCs in 6‐well plate as described in Basic Protocol 1, steps 39‐42.
23 Resuspend the cell pellet in EGM‐2 to obtain 2 × 10^6^ cells/ml. Pipet the cell suspension up and down gently once or twice with a 200‐µl pipet tip before injecting into each lumen.
  Ensure that the cell suspension is well mixed to achieve a consistent cell density between channels.
24 Take up 10 µl of cell suspension in a 10‐µl pipet tip. Insert the tip into the outlet medium reservoir with a light pressure.
25 Inject the cell suspension slowly and steadily until equal levels are reached across medium reservoirs and flow stops.
  Ensure that there is no air trapped inside the pipet tip, as the collagen scaffold can be deformed by injection of air bubbles. See Figure 3B illustrating how to inject the cell suspension correctly via medium reservoirs (I), and the examples of poor handling practice causing injection of air bubbles (III and IV).
26 Place the devices on a cell culture rotator (channel longitudinal axis in line with rotating axis). Rotate the devices for 1 hr at 1 rpm in the incubator at 37°C with 5% CO_2_.
27 Add 25 µl EGM‐2 to the outlet medium reservoir and 50 µl EGM‐2 to the inlet medium reservoir.
28 Dissociate hiPSC‐ECs in 6‐well plates from Support Protocol 2, step 12. Aspirate EC‐CGM and add 2 ml DPBS to each well.
29 Aspirate DPBS and add 0.5 ml TrypLE™ to each well. Incubate 3 min at room temperature.
30 Add 1.5 ml of EC‐SFM to each well to dilute TrypLE™. Transfer cell suspension to a 15‐ml tube using a 1000‐µl pipet tip. Pipet up and down gently once or twice.
31 Count the cells and centrifuge 3 min at 1100 rpm (300 × *g*) at room temperature. Aspirate the supernatant.
32 Resuspend the cell pellet in EGM‐2 supplemented with 50 ng/ml VEGF to reach 1 × 10^7^ cells/ml. Pipet the cell suspension up and down gently once or twice with a 200‐µl pipet tip before injecting into each lumen.
  The initial seeding density of ECs is critical for generating a confluent vessel barrier. Lower cell density results in gaps in the barrier (Fig. 3C, II and IV).
33 Remove any excess medium from medium reservoirs using a 10‐µl pipet tip. Leave ∼10 µl medium in the reservoir to avoid injecting air bubbles.
34 Take up 5 µl of hiPSC‐EC suspension in a 10‐µl pipet tip. Insert the tip into the outlet medium reservoir with a light pressure.
35 Inject the cell suspension into microchannels as described in step 25.
36 Place the devices on a cell culture rotator (channel longitudinal axis in line with rotating axis). Rotate the devices for 4 hr at 1 rpm inside the 37°C, 5% CO_2_ incubator.
37 Add 25 µl EGM‐2 supplemented with 50 ng/ml VEGF to the outlet medium reservoir and 50 µl to the inlet medium reservoir to create transient gravity‐driven flow (day 0).
  Refresh the vessel lumens daily with EGM‐2. The vessels can be used for functional assays or fixed on day 2 or 3.
38 Remove 30 µl EGM‐2 from each reservoir with 200‐µl pipet tip. Add 25 µl EGM‐2 to the outlet medium reservoir and 50 µl to the inlet medium reservoir to create transient gravity‐driven flow (days 1‐3).

## Support Protocol 3 STRUCTURAL CHARACTERIZATION OF 3D VoC MODEL

3D‐engineered vessels generated following Basic Protocol 2 can be characterized on day 2 or 3. Here we describe the structural characterization of the 3D vessels using immunofluorescence staining. The fixed vessels are first permeabilized for intracellular staining followed by blocking for at least 1 hr to avoid nonspecific binding of the primary antibody. In this protocol, we use primary antibodies against VE‐cadherin to visualize the EC cell junctions, and against contractile protein SM22 to visualize VSMC cell morphology. The secondary antibodies should be selected considering the host and target species of primary antibodies. The selection of specific fluorophores can vary between users and dilutions should be derived from manufacturer's recommendations. The stained 3D vessels are compatible with high‐resolution imaging using advanced microscopy.

*NOTE*: Always follow the same sequence for adding solutions into the vessel lumens (Basic Protocol 2, step 38).

### Materials

3D‐engineered vessels (Basic Protocol 2)
4% (w/v) PFA (see recipe)
0.1% (v/v) Triton X‐100 in DPBS (see recipe)
1% (w/v) BSA in DPBS (see recipe)
Dulbecco's phosphate‐buffered saline (DPBS; Life Technologies, cat. no. 14190)
Primary antibodies:
  Anti‐human VE‐cadherin, Rabbit, 1:200 (Cell Signaling, cat. no. 2158S)
  Anti‐human SM22 (TAGLIN/Transgelin), Rabbit, 1:200 (Abcam, cat. no. AB14106)

Parafilm sealing film, 10 cm × 38 m (Parafilm M, cat. no. 291‐1213)
Leica SP8 microscope, Dragonfly® spinning disk (Andor)
Imaris 9.5 software (Bitplane, Oxford Instruments)

1 Aspirate EGM‐2 from 3D vessels and add 4% (w/v) PFA to the medium reservoirs to fix the cells inside the lumens. Incubate 15 min at room temperature. Wash the lumens three times with DPBS, 10 min per wash, at room temperature.
  The fixed vessels can be stored in DPBS for up to 2 weeks at 4°C in a Parafilm‐sealed Petri dish. Prolonged storage increases the chance of evaporation and contamination.
2 Add 0.1% (v/v) Triton‐X 100 in DPBS to each lumen to permeabilize the cells. Incubate 15 min at room temperature.
3 Add 1% (w/v) BSA in DPBS to each lumen to block nonspecific binding sites. Incubate 1 hr at room temperature.
4 Dilute the primary antibodies in 1% (w/v) BSA in DPBS and add it to the corresponding lumens. Incubate overnight at 4°C.
  Primary antibodies used for characterization of vascular barrier and cell‐cell junctions include those for VE‐Cad and SM22.
5 Wash the lumens three times with DPBS, 15 min per wash, at room temperature.
6 Dilute the secondary antibodies in 1% (w/v) BSA in DPBS and add to the corresponding lumens. Incubate 2 hr at room temperature.
  The selection of secondary antibodies can vary between users, and the dilutions should be empirically determined depending on the specific antibody, incubation time, and temperature.
7 Wash the lumens three times with DPBS, 15 min per wash, at room temperature. Store the devices at 4°C in a Parafilm‐sealed Petri dish in the dark until microscopic imaging.
  The devices can be stored for prolonged periods in the dark and cold, although the highest‐quality images can be obtained within the first 1‐2 weeks. The reservoirs should be checked monthly for DPBS levels and refreshed regularly to prevent drying.

## REAGENTS AND SOLUTIONS

### l‐Ascorbic acid 2‐phosphate (AA2P), 5 mg ml^–1^

Add 250 mg AA2P (Sigma‐Aldrich, cat. no. A8960) to 50 ml dH_2_O, nonsterile. Prepare aliquots and store up to 1 year at −20°C.

### Basic fibroblast growth factor (FGF2) stock solution, 10 μg ml^–1^

Reconstitute at 100 μg ml^–1^ in distilled water (dH_2_O; Gibco™; Thermo Fisher Scientific, cat. no. 15230089). Further dilute to 10 μg ml^–1^ in 0.1% (w/v) BSA in DPBS (see recipe). Prepare aliquots and store up to 1 year at –80°C. Avoid repeated freeze‐thaw cycles.

### B(P)EL

Prepare 250 ml of B(P)EL using the volumes listed below. Sterilize medium using a Stericup filter and store up to 3 weeks at 4°C.

**Table:** 

**Composition**	**Volume**	**Final concentration**
IMDM	107.63 ml	
F12 nut. mix	113.88 ml	
PFHM‐II	12.5 ml	5%
10% (w/v) BSA/IMDM (see recipe)	6.25 ml	0.25%
Lipids (100×)	2.5 ml	1×
ITS‐X (100×)	250 μl	0.1×
αMTG (1.3%; see recipe)	750 μl	450 μM
AA2P (5 mg/ml; see recipe)	2.5 ml	0.05 m/ml
GlutaMAX (200 mM)	2.5 ml	2 mM
Pen/Strep (5000 U/ml)	1.25 ml	0.5%

IMDM, Iscove's modified Dulbecco's medium, no phenol red (Gibco™; Thermo Fisher Scientific, cat. no. 21056023); F12 nut. mix, Ham's F‐12 nutrient mix, GlutaMAX supplement (Gibco™; Thermo Fisher Scientific, cat. no. 31765027); PFHM‐II, Protein‐Free Hybridoma Medium, 1× (Gibco™; Thermo Fisher Scientific, cat. no. 12040077); Lipids, chemically defined lipid concentrate (Gibco™; Thermo Fisher Scientific, cat. no. 11905031); ITS‐X, insulin‐transferrin‐selenium‐ethanolamine (100×; Gibco™, Thermo Fisher Scientific, cat. no. 51500056); GlutaMAX, GlutaMAX‐1 supplement (Gibco™; Thermo Fisher Scientific, cat. no. 35050038); Pen/Strep, Penicillin‐Streptomycin (5000 U/ml; Gibco™; Thermo Fisher Scientific, cat. no. 15070063).

### BSA, 1% (w/v) in DPBS

Dissolve 0.5 g BSA (BovoStar BSA, Bovogen Biologicals, cat. no. BSAS 0.1) in 50 ml of DPBS on a bench shaker at cold room. Sterilize the solution by filtration using 0.22‐µm‐pore‐size membrane filter and store up to 4 weeks at 4°C.

### BSA, 10% (w/v) in IMDM

Add 5 g of BSA (BovoStar) to 40 ml of IMDM in a 50‐ml tube. Leave the tube for a few hours on a roller bank at 4°C until the BSA is dissolved completely. Add 10 ml IMDM to reach 10% (w/v) BSA final. Sterilize the solution by filtration with a 0.22‐μm‐pore‐size membrane filter and store for up to 4 weeks at 4°C.

### CHIR99021 (CHIR), 4 mM

Reconstitute CHIR99021 (Tocris Bioscience, cat. no. 4423) at 4 mM in DMSO. Prepare aliquots and store up to 1 year at −20°C.

### Citric acid, 5 mM (pH ∼3)

Add 960.6 mg citric acid (Sigma‐Aldrich, cat. no. 27487) to 1 liter dH_2_O. Sterilize the solution by filtration with a 0.22‐μm‐pore‐size membrane filter and store up to 3 months at 4°C.

### Collagen I stock solution, 10 mg/ml

Prepare 200‐μl aliquots of 10 mg/ml collagen I from rat tail collagen stock solution (Ibidi, cat. no. 50204). Store the aliquots according to the manufacturer's instructions.

The high viscosity of the stock solution can cause pipetting errors during aliquoting. Before proceeding to the dilution step, it is highly advisable to weigh the collagen I stock aliquots to verify the quantity.

### Collagen I solution, 5 mg/ml (pH ∼7.4)

**Table:** 

**Mixing order**	**Component**	**Volume (μl)**
i	10× M199	42.7
ii	dH_2_O	142.8
iii	7.5% (w/v) NaHCO_3_	12.5
iv	1 M NaOH	2
v	10 mg/ml collagen I stock (see recipe)	Weight (mg) 200

10× M199 and 7.5% sodium bicarbonate (NaHCO_3_) solution are from Gibco™ (Thermo Fisher Scientific, cat. nos. 11825015 and 25080060) and 1 M sodium hydroxide (NaOH) from Merck (cat. no. 1064981000).

### Cut 10‐μl pipet tips, 7 mm (for VFP)

Cut off the tip of 10‐μl pipet tips (Greiner Bio‐One, cat. no. 741015) 7 mm from the tip using a razor blade. Autoclave the cut pipet tips at least 3 days before experiment.

A custom‐fabricated cutting guide is used to obtain consistent length.

### Endothelial Cell Complete Growth Medium (EC‐CGM)

Prepare EC‐CGM using the volumes listed below. Sterilize the medium with a Stericup filter and store up to 2 weeks at 4°C.

**Table:** 

**Composition**	**Volume**	**Final concentration**
EC‐SFM	247.5 ml	
hPPS	2.5 ml	1%
VEGF, 50 μg/ml	150 µl	30 ng/ml
bFGF, 100 μg/ml	50 µl	20 ng/ml

EC‐SFM, Invitrogen, cat. no. 11111‐044); hPPS, human serum from platelet‐poor plasma (hPPS; Sigma‐Aldrich, cat. no. P2918); VEGF, human VEGF (165) IS, premium grade (Miltenyi Biotec, cat. no. 130‐109‐386); bFGF, human bFGF, premium grade (Miltenyi Biotec, cat. no. 130‐093‐842).

### EDTA, 0.5 M (pH 8.0)

Add 186.1 g EDTA disodium salt (Sigma‐Aldrich, cat. no. E5134) to 800 ml dH_2_O. Adjust the pH to 8.0 with NaOH and add dH_2_O to a final volume of 1 liter. Filter the solution through a 0.22‐μm‐pore‐size membrane filter and sterilize by autoclaving. Store up to 1 year at room temperature.

### EGM‐2

Prepare a 500‐ml bottle of EGM‐2 as described by the manufacturer, adding all supplements from the kit to the basal medium. Additionally, add 2.5 ml Penicillin‐Streptomycin (5000 U/ml; Gibco™; Thermo Fisher Scientific, cat. no. 15070063). Store the medium up to 6 weeks at 4°C.

### Fibronectin‐coated 96‐well plate

Dilute fibronectin (Sigma‐Aldrich, cat. no. F1141) in DPBS to final concentration of 5 μg/ml. Add 100 μl to each well of 96‐well plate. Incubate at room temperature for at least 1 hr before use. Stored up to 2 weeks at 4°C.

### Fluorescence‐activated cell sorting buffer (FACSB)

Dissolve 1.25 g BSA in 250 ml DPBS and add 1 ml of 0.5 M EDTA (pH 8.0; see recipe). Sterilize using a Stericup filter (0.22 μm pore size) and store up to 4 weeks at 4°C.

### Gelatin‐coated 6‐well plate

Add 0.1% (w/v) gelatin solution (see recipe) to a culture plate or flask. Ensure that the gelatin covers the whole surface. Use 1 ml gelatin solution per well of a 6‐well plate. Leave the plate with gelatin for 1 hr at 37°C before use. The coated plates can be stored up to 2 weeks at 4°C.

### Gelatin solution, 0.1% (w/v)

Add 1 g gelatin (from porcine skin, type A; Sigma‐Aldrich, cat. no. G1890) to 100 ml of dH_2_O to prepare a 1% (w/v) gelatin solution. Autoclave the solution and store 10‐ml aliquots up to 1 year at −20°C.

For 0.1% (w/v) gelatin working solution, dilute stock solution 10× with Dulbecco's phosphate‐buffered saline (DPBS; Life Technologies, cat. no. 14190). Sterilize the solution by filtration with a 0.22‐μm‐pore‐size membrane filter and store up to 1 year at room temperature.

### Matrigel‐coated 6‐well plate

Thaw 0.5 mg Matrigel (Growth Factor Reduced; Corning, cat. no. 354230) on ice for each 6‐well culture plate. Aliquot 6 ml cold (4°C) Dulbecco's modified Eagle's medium/nutrient mixture F‐12 (DMEM/F12) medium into a 50‐ml tube. Cool a pipet tip by pipetting up and down the cold DMEM/F12 medium several times and use it to transfer thawed Matrigel into the medium. Mix with a cold (4°C) 5‐ml pipet and add 1 ml to each well. Leave the plate at room temperature for 1 hr. Use the plate right away or seal with Parafilm and store up to 2 weeks at 4°C.

### Medium reservoirs for microchannels (cut 1000‐μl pipet tips)

Cut 1000‐μl pipet tips (Greiner Bio‐One, cat. no. 740295) ∼20 mm from the tip using razor blade. Autoclave the cut pipet tips at least 3 days before experiment.

### α‐Monothioglycerol (αMTG) solution, 1.3% (v/v)

Add 130 μl mono‐thio glycerol (α‐MTG; Sigma‐Aldrich, cat. no. M6145‐25 ml) to 9.87 ml Iscove's modified Dulbecco's medium (IMDM) and store up to 1 year at 4°C protected from light.

### Neural crest (NC) differentiation medium

Prepare neural crest differentiation medium using the volumes listed below. Always prepare fresh.

**Table:** 

**Composition**	**Volume**	**Final concentration**
B(P)EL (see recipe)	4 ml	
SB431542 (20 nM; see recipe)	2 μl	10 µM
CHIR99021 (4 mM; see recipe)	1 μl	1 µM
FGF2 (10 μg/ml; see recipe)	4 μl	10 ng/ml

### PDGF‐BB stock solution, 20 μg/ml

Reconstitute PDGF‐BB (PeproTech, cat no. 100‐14B) at 20 μg/ml in 0.1% (w/v) BSA in DPBS. Prepare aliquots and store at –20°C or below for up to 1 year. Avoid repeated freeze‐thaw cycles.

### PFA, 4% (w/v)

Mix 1 vol of 8% (w/v) PFA (see recipe) with 1 vol of 0.2 M phosphate buffer (pH 7.4; see recipe). Keep for up to 2 weeks stored at 4°C.

CAUTION: Work in a fume hood.

### PFA, 8% (w/v)

Add 40 g paraformaldehyde (PFA; Merck, cat. no. 1.04005.1000) to 400 ml Milli‐Q water. Heat to 60°C and stir it at medium speed. After a few minutes, add ∼10 drops of 1 N NaOH to dissolve the PFA granules. Eventually, the solution will become translucent. Let the solution cool down and add Milli‐Q water to a total volume of 500 ml. Store the solution up to 2 months at 4°C.

CAUTION: Work in a fume hood.

### Phosphate buffer, 0.2 M (pH 7.4)

To prepare solution 1, dissolve 8.28 g sodium dihydrogen phosphate dihydrate (NaH_2_PO_4_·2H_2_O; J.T. Baker, cat. no. 0326) in 300 ml Milli‐Q ultrapurified water (e.g., from a Millipore system). To prepare solution 2, dissolve 10.78 g disodium hydrogen phosphate monohydrate (NaH_2_PO_4_·1H_2_O; Merck, cat. no. 1.06346.1000) in 300 ml Milli‐Q water. Add solution 1 to solution 2 until a pH of 7.4 is obtained, to make 0.2 M phosphate buffer (pH 7.4). This buffer can be stored at room temperature indefinitely (no expiration date).

### Polydopamine (PDA), 2 mg/ml (w/v)

Dissolve dopamine hydrochloride (Merck, cat. no. H8502‐5G) in 10 mM Tris·Cl buffer (pH 8.5; see recipe) to obtain 2 mg/ml (w/v) polydopamine (PDA) solution. Filter the buffer through a 0.22‐μm‐pore‐size membrane filter. Always prepare fresh.

### SB43152 solution, 20 mM

Reconstitute SB43152 (Tocris Bioscience, cat. no. 1614) at 20 mM in DMSO. Prepare aliquots and store up to 1 year at −20°C.

### Transforming growth factor beta‐3 (TGF‐β3) stock solution, 5 μg/ml

Reconstitute TGF‐β3 (PeproTech, cat. no. 100‐36E) at 100 μg/ml in 5 mM citric acid (see recipe). Further dilute to 5 μg/ml in 0.1% (w/v) BSA in DPBS (see recipe). Prepare aliquots and store up to 1 year at −20°C.

### Tris·Cl buffer (pH 8.5), 10 mM

Dissolve 1.2 g/liter Tris (Trizma®) base (Tris(hydroxymethyl)aminomethane; Sigma‐Aldrich, cat. no. T6066‐1KG) and 1.0 g/liter sodium azide (Sigma‐Aldrich, cat. no. S8032‐25G) in ∼90% final volume with cold dH_2_O. Add cold dH_2_O to obtain final volume. Adjust to pH 8.5 with 5 M HCl or 5 M NaOH as needed. Filter the buffer through a 0.22‐μm‐pore‐size membrane filter. Store up to 4 years at room temperature.

### Triton X‐100, 0.1% (v/v) in DPBS

Add 250 µl Triton X‐100 (Sigma‐Aldrich, cat. no. T8787) to 50 ml DPBS. Sterilize the solution by filtration using a 0.22‐µm‐pore‐size membrane filter. Store up to 1 year at room temperature.

### Vascular endothelial growth factor (VEGF) stock solution, 50 μg/ml

Reconstitute human VEGF (165) IS, premium grade (Miltenyi Biotec, cat. no. 130‐109‐386) at 50 μg ml^–1^ in 0.1% (w/v) BSA in DPBS. Prepare aliquots and store up to 1 year at −80°C.

### Vitronectin‐coated 6‐well plates

Prewarm Cell Adhere dilution buffer (Stemcell Technologies, cat. no. 07183) to room temperature. For each well of a 6‐well plate, thaw 40 μl VitronectinXF (Stemcell Technologies, cat. no. 07180) at room temperature and add to 1.21 ml dilution buffer. Mix well and add 1.25 ml per well to the wells of a 6‐well cell suspension plate. Distribute vitronectin to cover the whole well and incubate at room temperature for 1 hr. Use the plate right away or seal with Parafilm and store up to 2 weeks at 4°C.

### Vascular smooth muscle cell (VSMC) differentiation medium

Prepare VSMC differentiation medium using the volumes listed below. Always prepare fresh.

**Table:** 

**Composition**	**Volume**	**Final concentration**
B(P)EL (see recipe)	25 ml	
TGFβ3 (5 µg/ml; see recipe)	10 μl	2 ng/ml
PDGFBB (20 µg/ml; see recipe)	12.5 μl	10 ng/ml

## COMMENTARY

### Background Information

Since the initial discovery of hiPSCs, directed differentiation protocols to generate vascular cell types under defined conditions have significantly improved. The potential applications of hiPSC‐derived vascular cells are broad and include engineering of human blood vessels and the study of vascular‐disease‐related abnormalities. Developmentally, VSMCs originate from multiple lineages including mesoderm and NC, the main source of VSMCs in the cerebral vasculature (Majesky, 2007). Several protocols have reported methods to induce NC formation in hiPSC using different chemical cocktails based on the factors that target bone morphogenic protein, WNT, and fibroblast growth factor signaling. Our protocol described here induces NC formation by combining an inhibitor of glycogen synthase kinase 3, CHIR99021, to activate canonical WNT signaling together with basic fibroblast growth factor and the activin/nodal inhibitor SB431542. After 12 days of culture, more than half of the cells become CD271^+^ and can then be cryopreserved and thawed for further differentiation and expansion. At P3, hiPSC‐NCCs derived from this protocol show a high total CD271^+^ NCC yield and high reproducibility across different hiPSC lines and batches. Importantly, these hiPSC‐NCCs preserve their phenotype up to P7, which provides a distinct advantage for the generation of large cell batches for disease modelling in vitro. To induce VSMC differentiation from hiPSC‐NCCs, we used PDGF‐BB and TGF‐β3 for another 12 days, based on previously described protocols (Cheung et al., 2012; Dash et al., 2016; Granata et al., 2016; Halaidych et al., 2019a; Wanjare et al., 2013, 2014). hiPSC‐VSMCs can be cryopreserved on day 8 and later thawed for further differentiation until day 12. At this stage, hiPSC‐VSMCs exhibit increased expression of the contractile proteins calponin 1, α‐smooth muscle actin (αSMA), and SM22, as previously described (Halaidych et al., 2019a). This protocol has the particular advantage that it successfully induces hiPSC‐VSMC differentiation and upregulation of contractile proteins and is based on serum‐free (defined) medium. To assess hiPSC‐VSMC functionality, we developed an automated pipeline that includes the determination of intracellular Ca^2+^ release and contraction in response to vasoconstrictors. Specifically, this defines a robust approach for quantification and specification of VSMC function by providing multiple parameters to accurately assess phenotype in the overall cell population across hiPSC‐VSMCs differentiated from different hiPSC lines (Halaidych et al., 2019b).

Additionally, we previously described a protocol to efficiently induce EC differentiation from hiPSCs through mesoderm specification under defined culture conditions (Orlova et al., 2014b). We carried out extensive functional characterization of these hiPSC‐ECs using multiple assays, including a comparative assessment of barrier function and inflammatory responses upon treatment with various proinflammatory agents. We further demonstrated the capacity of stimulated hiPSC‐EC monolayers to recruit cells of the human monocytic cell line THP1 (Halaidych et al., 2018), blood monocytes, and hiPSC‐derived monocytes (Cao et al., 2019, 2020) by using 2D microfluidic flow assays. Overall, we can robustly produce large quantity of hiPSC‐derived VSMCs and ECs that are fully functional after cryopreservation. Recently, we demonstrated the potential utility of these hiPSC‐derived ECs and VSMCs by developing a robust multicellular 3D VoC model that supports the formation of a functional self‐assembled microvasculature network within fibrin hydrogel inside a microfluidic chip (Vila Cuenca et al., 2021).

Current VoC models based on the self‐organization of vascular cells into microvasculature networks are of great value for studying physiological aspects of vascular development in vitro. However, the complex and intrinsically variable architecture makes it challenging to precisely control fluid flow and the wall shear stress exposed to the vascular barrier during dynamic flow assays. To address this issue, alternative VoC platforms are being developed to fabricate controlled and defined vascular architectures. One widely used approach is the insertion of removable templates (e.g., a small‐diameter needle or silicon rod) inside an unpolymerized hydrogel to create 3D lumen scaffolds that are subsequently used for cell culture (Hasan, Paul, Memic, & Khademhosseini, 2015; Jiménez‐Torres et al., 2015). Although it is straightforward, this method carries certain limitations: it is laborious, time consuming, and challenging to scale up. In 2012, Bischel et al. reported a practical approach for generating lumen scaffolds inside microfluidic channels by pipetting only fluids. The method exploits the viscosity difference between two fluids: less viscous fluid (cell culture medium) flows through and displaces more viscous fluid (unpolymerized hydrogel), leaving a hollow structure upon polymerization (Bischel, Lee, & Beebe, 2012). The method was applied to pattern a variety of controlled geometries, including multiple parallel lumens, to study vascular barrier function and angiogenesis (Bischel, Young, Mader, & Beebe, 2013). The lumen structures supported the multicellular culture of primary ECs, pericytes, and astrocytes with the aim of modeling blood‐brain barrier (BBB) in vitro (Herland et al., 2016).

In our recent work, we implemented the VFP technique and optimized it further to improve the reproducibility of patterning uniform lumen scaffolds in collagen gel. We extended the patterning pathway using a cut 10‐μl pipet tip on the outlet port to create a constant driving force for patterning. An extension length of 7 mm was found to be optimal to achieve a uniform intra‐ and interlumen diameter profile, giving reproducible results in the context of dynamic functional studies (de Graaf et al., 2019). Before collagen patterning, surface functionalization of PDMS is required to promote the strength of adhesion to collagen I hydrogel. In this protocol, we use a single‐step functionalization technique based on polydopamine (PDA; Park, Georgescu, Oh, Kwon, & Huh, 2019). Alternative methods for surface functionalization have been reported previously that can be implemented prior to collagen patterning using the VFP protocol (de Graaf et al., 2019; Polacheck, Kutys, Tefft, & Chen, 2019; Yadav, Sriram, Carter, & Miller, 2014). Our VFP protocol has particular advantages compared to other approaches, including its robustness, high reproducibility between experiments, low cost, and potential for scale‐up without the need for specialized equipment, as dozens of lumen scaffolds can be efficiently patterned in a few minutes.

Here, we also describe the successful integration of hiPSC‐VSMCs and hiPSC‐ECs into patterned collagen lumen scaffolds in a sequential manner. 3D vessel structures are efficiently formed in a couple of days that reveal close interactions of hiPSC‐ECs and hiPSC‐VSMCs, which is crucial for recapitulating physiological EC‐mural cell crosstalk found in vivo (Gaengel et al., 2009). In our experience, 3D vessels can be stable for up to 7 days; however, long‐term culture is challenging and is impractical for dynamic studies. The cells remodel collagen gel and over time the lumen scaffold becomes deformed over, mostly because of the high proliferation of hiPSC‐VSMCs. Another limitation is that the model is not optimal for high‐throughput studies such as drug screening, as the time required for manual fabrication of PDMS devices limits truly mass production. Overall, 3D multi‐cell‐type VoC model generated using this protocol can potentially be used in many applications integrating hemodynamic and mechanical parameters for physiological vessel function. Using hiPSCs derived from patients with specific disease genotypes presents opportunities for applications that include, but are not limited to, cardiovascular and brain vascular disease modeling.

### Critical Parameters

hiPSCs used for differentiation in this protocol are expanded on vitronectin‐coated plates in defined TeSR‐E8 medium. It is important to limit the spontaneous differentiation of hiPSC colonies in culture for a robust differentiation. To successfully maintain the pluripotent state, the colonies should be passaged regularly every week. Before seeding, large colonies should be fragmented into small aggregates (100‐200 µm) by pipetting to increase their adherence and survival. Compact colonies with >90% confluency should be achieved after 1 week of passaging. Initial seeding density of hiPSCs is a crucial step for efficient NC differentiation. The recommended split ratio is 1:30 or 1:40 from a well that is >90% confluent; this will result in 20‐30 hiPSC colonies attached the next day. However, the split ratio may need further optimization, depending on the line, to achieve similar results. On day 0, before NC induction, the colonies should be 200‐400 µm in size. As differentiated hiPSC‐NCCs start migrating from the colony edges on day 5‐7, mechanical elimination of the cells in the colony centers becomes essential for efficient differentiation (Fig. 1A; day 8a and day 8b). hiPSC‐NCCs should be further differentiated and expanded to P3 before VSMC induction. The initial density of hiPSC‐NCCs is crucial for efficient VSMC differentiation (day 0, Fig. 1C). It is best to cryopreserve hiPSC‐VSMCs on day 8 and to prepare a batch of cells from the same differentiation that is sufficient for multiple experiments.

The generation of 3D‐engineered vessels consists of three consecutive parts: fabricating and assembling the PDMS devices, patterning the collagen I lumen scaffold, and integrating the hiPSC‐VSMCs and hiPSC‐ECs in a sequential manner. Microfluidic devices are fabricated by casting PDMS on SU‐8 master mold. The inlet and outlet ports should be opened by using a biopsy puncher with a diameter of 1.2 mm, which is an optimal size for inserting pipet tips, reservoirs, and tubing of external microfluidic pumps. PDMS devices can be stored in a dry, dust‐free place and should be cleaned with Scotch tape before assembly. Air‐plasma treatment provides a transient change in the property of the surface by rendering it hydrophilic for a short time, and therefore treated surfaces should be contact bound immediately. Pressing gently after bonding ensures that there is no air trapped in between surfaces. In this protocol, we use PDA for surface functionalization, which does not require freshly plasma‐treated surfaces. One possible drawback is the slight darkening of PDMS if high concentrations of PDA are used or in the case of a prolonged incubation time. The final concentration of 2 mg/ml PDA with a 1‐hr incubation hr gives efficient results and no interference with the optical transparency of PDMS. Before patterning, the channels should be dried completely for efficient collagen attachment.

The collagen gel preparation contains critical steps concerning the final concentration, pH, and temperature, which particularly affect the success rate and the lumen diameter. In this method, we recommend using highly concentrated collagen I derived from rat tail. The specifications of the stock collagen I solution can vary between different batches and manufacturers. The calculations should be empirically determined following the manufacturer's instructions for each different batch. To improve the reproducibility in this step, we verify the exact quantity of each collagen I aliquot by weight. The volumes of each reagent should be adjusted according to the stock concentration and the final weight of the collagen aliquot. The mechanical properties of collagen fibers re greatly influenced by the final pH and polymerization temperature. The pH of the collagen solution should be adjusted to ∼7.4; acidic or alkaline collagen solutions should not be used for patterning as that will dramatically affect the success rate and lumen diameter. All reagents should be always kept ice cold to delay the polymerization of collagen fibers. Patterning fails dramatically when partially polymerized collagen gel is used. Therefore, we recommend limiting the procedure time to 5 min per batch of mixed collagen. Consistency is the key to generating reproducible lumen scaffolds that create constant physiological conditions for culturing vascular cells. A uniform lumen diameter profile between experiments is achieved by creating a constant driving force for patterning. For this, we insert the 10‐μl pipet tips of consistent 7‐mm length into the outlet ports. After patterning, the pipet tips need to be removed by twisting to allow access to the lumen scaffolds for cell seeding.

Air bubbles are the most common reason for lumen deformation during the cell seeding step. However, this can be avoided if the cell seeding step is carried out correctly (Fig. 3B and 3I). We illustrate the potential ways in which air bubbles may be injected in Figure 3B, III and IV; we recommend checking each port for air bubbles and removing it if needed (Fig. 3B, II) before proceeding with cell seeding. Equilibrating cell culture medium helps to reduce the emergence of air bubbles over time due to the temperature differences. We found that injection of small air bubbles during cell seeding can enlarge lumen scaffolds. To eliminate variations in diameter, cells should be injected slowly and steadily. The initial cell seeding density is absolutely crucial for the formation of a confluent 3D vessel barrier (Fig. 2D). Low seeding densities of hiPSC‐ECs fail to form a confluent endothelial barrier (Fig. 3C, II and IV). High seeding densities of hiPSC‐VSMCs can induce deformation of collagen scaffold in time due to the cells’ high proliferation. Inconsistency in seeding densities between channels can be avoided by keeping the cell suspension homogeneous. After cell seeding, the liquid levels in opposite reservoirs should be equilibrated to stop the flow of cells. Rotating the devices promotes even distribution of the cells along the collagen lumen surface. Compared to the protocol published previously (de Graaf et al., 2019), we reduced the time for which the devices need to be rotated after hiPSC‐EC seeding to 4 hr, which is optimal for hiPSC‐EC attachment to lumen scaffolds. The rotation time might need to be optimized for ECs and mural cells from other sources. The 3D vessels should be refreshed every day due to the small volume of cell culture medium, a total of 75 µl per channel. Severe evaporation of the medium can be prevented by placing water in a dish next to the devices. Care should be taken to avoid injecting air bubbles when refreshing the vessels as discussed before for cell seeding. A transient flow is induced during daily refreshments by creating a hydrostatic pressure difference across medium reservoirs. This should be consistent for each refreshment or washing step.

### Troubleshooting

Detailed troubleshooting guidelines can be found in Table 1.

**Table 1:** Troubleshooting

Step(s)	Problem(s)	Possible cause(s)	Solution(s)
Basic Protocol 1, step 12 or 16	Low efficiency of NC differentiation; low % CD271^+^SOX2^–^ and high % CD271^–^SOX2^+^	Starting seeding density of hiPSCs was not optimal	Ensure that 20‐30 hiPSC colonies 200‐400 µm in size are present on day 0, before start of differentiation.
		Colony centers were not eliminated sufficiently	Mechanically eliminate all colony centers on day 8 or 9 (Fig. 1A; days 8a and 8b).
Basic Protocol 1, step 34	Low efficiency of VSMC differentiation	Initial seeding density of hiPSC‐NCCs was not optimal	Seed ∼2.5 × 10^5^ hiPSC‐NCCs in each well of a 6‐well plate.
Basic Protocol 2, steps 9‐10	Dark PDMS channels after surface functionalization	Final concentration of PDA solution was too high, or incubation duration was too long.	Reduce the final concentration of PDA solution or the duration of incubation.
		Washing after PDA incubation was insufficient	Wash the microchannels thoroughly with dH_2_O.
Basic Protocol 2, step 13	Inconsistent pH values between batches for the final collagen solution	Calculations for the preparation of the final collagen solution were wrong	Weigh the stock collagen I aliquots to verify the quantity. Update the volume of each reagent according to the quantity and concentration of stock aliquots.
Basic Protocol 2, steps 13‐15	Inconsistent lumen diameter and uneven geometry; partially patterned lumens	Driving force created for patterning was inconsistent	Ensure that all cut 10‐μl pipet tips are 7 mm long. Use a cutting guide to improve consistency. Introduce adequate volume of driving fluid (3 µl of DPBS) on top of the cut pipet tip to create stable driving force.
		Preparation of collagen solution was inconsistent	Check the calculations for collagen final solution. Weigh the collagen to verify quantity. Ensure the final pH is ∼7.4 and the solution is pink in color.
		Partially polymerized collagen solution was used for patterning	Keep all reagents on ice during collagen preparation. Use the neutralized collagen solution within 5 min. Immediately place the patterned channels in the incubator at 37°C.
		Collagen stock is old	Store high‐concentration collagen I stock solution as recommended and ≤4 months.
Basic Protocol 2, step 9	Delamination of collagen lumen scaffold during cell seeding or culture	Surface functionalization was inefficient	Optimize concentration and incubation time for PDA treatment. Thoroughly the PDMS channels and dry completely with pressured air.
Basic Protocol 2, step 25		Air bubbles are injected into the lumens	Equilibrate the cell culture medium; inspect the channel ports before the injection step and remove any air bubbles (Fig. 3B, II).
Basic Protocol 2, steps 23‐25 and 32	Inconsistent cell seeding density	Cell suspension was not mixed well before seeding	Pipet the cell suspension up and down before injecting into each lumen.
		Seeded cells in the lumens are flushed out due to gravity‐driven flow	Equilibrate the medium levels across reservoirs to stop the flow of cells in the lumens.

### Understanding Results

Initial seeding density of hiPSCs is a crucial step for efficient NC differentiation. In our experience, low‐density cultures yield higher differentiation efficiency. A yield of 20‐30 attached hiPSC colonies is expected the next day. These colonies expand continuously from day –2 to day 4, reaching 200‐400 µm in size on day 0 of NC induction. Differentiated hiPSC‐NCCs should start migrating outside the colonies from around day 5‐7, and their number should grow continuously until day 12. On day 12 (P0), >50% of the cells are expected to be CD271^+^ hiPSC‐NCCs. A low percentage of CD271^+^ cells indicates inefficient differentiation or mechanical elimination of colony centers (Fig. 1A; day 8a and day 8b). An enriched cell population with >90% CD271^+^ cells should be obtained for hiPSC‐NCCs at P3 (Fig. 1B). In our experience, the phenotype is maintained up to P7. Both fresh or cryopreserved hiPSC‐NCCs (P3) can be used to differentiate VSMCs. To induce VSMC differentiation, ∼2.5 × 10^5^ hiPSC‐NCCs should be seeded to each well of the 6‐well plate (day –1). The next day, hiPSC‐NCCs should have a density as shown in Figure 1C before the start of VSMC differentiation (day 0). Until day 8, cells will expand and acquire a bigger and spindle‐like morphology (Fig. 1C). A confluent monolayer should be observed before passaging (day 4) and cryopreservation (day 8). Thawed hiPSC‐VSMCs should continue further differentiation and reach >90% confluency 4 days after thawing (day 12, Fig. 2A). hiPSC‐VSMCs should express the contractile markers SM22, calponin 1 (CNN1), and αSMA (ACTA2) on day 12 (Fig. 1D). Optionally, functional assays can be performed to further characterize the hiPSC‐VSMCs (Halaidych et al., 2019a, 2019b).

Microfluidic devices are fabricated by casting PDMS and assembled with PDMS‐coated cover glasses creating watertight microchannels. The efficiency of the contact bonding can be checked by slightly lifting the device or injecting a solution into the microchannels. Surface functionalization with PDA can cause slight darkening of PDMS, but this is negligible under the conditions we use in this protocol. Inefficient functionalization can cause the detachment of collagen I scaffold from the PDMS surfaces. Sterilization of devices with UV and ozone is effective against contamination and does not interfere with PDA treatment. Before patterning, a stock collagen I aliquot (10 mg/ml) with a defined weight is diluted to 5 mg/ml final following the recipe in Reagents and Solutions. The pH is estimated by the color of the phenol red from the 10× M199 medium, which is expected to be pink, indicating a pH of ∼7.4 (Fig. 2C and 2I). Acidic gel solutions (yellow) can be adjusted by adding NaOH dropwise until the color changes to pink. Alkaline solutions (purple) should be discarded. The patterning is initiated by introducing a droplet of DPBS (3 µl) into the cut 10‐μl pipet tip (outlet), which immediately flows through the collagen gel and disappears. Any DPBS remaining on the cut 10‐μl pipet tip is an indication of failed patterning. The VFP protocol can be further scaled up using a syringe to inject collagen I and a multi‐dispenser pipet to add DPBS droplet. In this way, 24 channels can be easily patterned within 5 min. After removal of the pipet tips next day, the patterned lumens are expected to be perfusable and accessible via the ports. The structure of 3D lumens and collagen fibers can be visualized using the two‐photon second‐harmonic generation (2P‐SHG) technique without the need to stain the collagen fibers (Fig. 2C, III and IV). Additionally, perfusability of the lumens can be quickly assessed by flowing fluorescent beads.

Thawed hiPSC‐VSMCs and hiPSC‐ECs are prepared for VoC culture in 4 days (Fig. 2A). Representative images of sequential cell‐seeding steps with optimal cell densities, using genetically targeted hiPSC‐VSMCs (ACTB; green) and hiPSC‐ECs (mCherry; red), are shown in Figure 2D. First, hiPSC‐VSMC suspension is injected in the lumens via the inlet medium reservoir (Fig. 2D and 2I). The collagen lumen scaffold is evenly covered with attached hiPSC‐VSMC and the devices are rotated on a cell culture rotator for 1 hr (Fig. 2D, II). Next, hiPSC‐EC suspension is injected into the lumens (Fig. 2D, III) and the devices are rotated for 4 hr. The next day (day 1), a confluent hiPSC‐EC layer is formed, with surrounding hiPSC‐VSMCs spreading and covering the hiPSC‐EC layer in the following days. The hiPSC‐EC layer remains stable, while hiPSC‐VSMCs continue proliferating and maintain cell‐cell interaction with hiPSC‐ECs. Success rate of vessel formation in lumen scaffolds is >90%, and the resulting diameter is consistent between experiments, averaging 298 ± 17.82 μm (*n* = 12; *N* = 4) on day 3. In our experience, wider lumen diameters are expected after cell seeding as the lumen scaffolds can easily be deformed by high pressures or air bubbles during cell seeding (de Graaf et al., 2019). 3D‐engineered vessels can be used for functional and dynamic assays on day 2 or 3 under physiologically relevant wall shear stress. Using genetically targeted hiPSC lines, the cell‐cell interactions can be tracked in real time. The structure of 3D vessels can be visualized by immunofluorescence analysis (Support Protocol 3) and cell‐cell interaction can be assessed using high‐resolution fluorescence microscopy. 3D reconstruction reveals a continues hiPSC‐EC barrier with integral VE‐Cadherin junctions (red) and closely interacting SM22 (green) positive hiPSC‐VSMCs (Fig. 4).

### Time Considerations

#### Basic Protocol 1

Differentiation of NCCs from hiPSCs takes 12 days in total:

Steps 1‐8: ∼20 min to passage hiPSCs for maintenance;

Steps 9‐13: ∼20 min to passage hiPSCs to a Matrigel‐coated plate for NC differentiation;

Step 15: ∼15 min every 2 days to refresh the cells;

Steps 16‐17: ∼15 min to detach the centers of colonies and refresh each well.

Differentiation and expansion of hiPSC‐NCCs to P3 take 8‐11 days in total:

Steps 19‐25: ∼20 min to passage hiPSC‐NCCs for expansion;

Steps 27‐30: ∼30 min to cryopreserve hiPSC‐NCCs.

Differentiation of VSMCs from hiPSC‐NCCs takes 12 days:

Steps 31‐34: ∼20 min to thaw cryopreserved hiPSC‐NCCs;

Steps 36‐37: ∼15 min every 2 days to change the medium;

Steps 38‐44: ∼20 min to passage cells;

Steps 45 and 47: ∼15 min to refresh the cells;

Steps 48‐51: ∼30 min to cryopreserve hiPSC‐VSMCs.

#### Support Protocol 1

Analysis of hiPSC‐NCCs by flow cytometry takes 1 day:

Steps 1‐11:∼2.5 hr to perform surface staining followed by intracellular staining of hiPSC‐NCCs;

Step 12: ∼30 min are needed to run the samples by flow cytometry.

Immunofluorescence staining of hiPSC‐VSMCs takes 2 days:

Steps 13‐15: ∼20 min to dissociate and seed hiPSC‐VSMCs followed by 24 hr culture;

Steps 16‐19: ∼1.5 hr to fix, wash, permeabilize, and block the cells;

Step 20: ∼ 15 min to dilute primary antibodies and add to them the wells;

Steps 21‐22:∼45 min to wash the cells and add diluted secondary antibodies;

Step 23: ∼45 min to wash the cells.

#### Support Protocol 2

Further differentiation of hiPSC‐VSMCs takes 4 days:

Steps 1‐5: ∼20 min to thaw cryopreserved cells and seed into the wells;

Step 6: ∼15 min to refresh on day 10.

Expansion of hiPSC‐ECs takes 4 days:

Steps 7‐11: ∼20 min to thaw cryopreserved cells and seed into the wells;

Step 12: ∼15 min to change the medium on day 3.

#### Basic Protocol 2

The fabrication of PDMS devices takes 2 days:

Steps 1‐3: ∼20 min to prepare PDMS followed by overnight curing;

Step 4: ∼20 min to coat the cover glasses with PDMS followed by overnight curing;

Steps 5‐8: ∼30 min to cut and assemble PDMS devices;

Step 9: ∼1.5 hr min to functionalize PDMS surface, including 1 hr PDA incubation;

Step 10: ∼1 hr to run UV + ozone sterilization.

Patterning of collagen I lumen scaffolds takes 2 days:

Step 12: ∼5 min to insert pipet tips into the inlet and outlet ports;

Steps 13‐14: ∼5 min to prepare the collagen gel;

Step 15: ∼5 min to pattern the lumens followed by 30 min for collagen polymerization;

Step 16: ∼5 min to add medium on polymerized collagen gel followed by overnight incubation.

Cell seeding takes 1 day, with 2 hr total active work and 5 hr for rotation of the devices:

Steps 17‐20: ∼10 min to replace pipet tips with medium reservoirs;

Steps 21‐26: ∼30 min to dissociate hiPSC‐VSMCs, count and seed into the lumens followed by 1 hr rotation;

Step 27: ∼5 min to add medium into the lumens;

Steps 28‐36: ∼30 min to dissociate hiPSC‐Ecs and count and seed them into the lumens followed by 5 hr rotation;

Steps 37‐38: ∼10 min to refresh the lumens every day.

#### Support Protocol 3

Immunofluorescence staining of vessels takes 2 days:

Steps 1‐3: ∼1.5 hr min to fix, permeabilize, and block the cells;

Step 4: ∼15 min to dilute primary antibodies and add to the lumens;

Steps 5‐6: ∼45 min to wash the lumens and add diluted secondary antibodies followed by 2 hr incubation;

Step 7: ∼45 min to wash the lumens.

### Author Contributions

**Merve Bulut**: Writing─original draft; **Marc Vila Cuenca**: Writing─original draft, writing─review & editing; **Mees de Graaf**: Methodology; **Francijna E. van den Hil**: Writing─original draft, writing─review & editing; **Christine L. Mummery**: Funding acquisition, writing─review & editing; **Valeria V. Orlova**: Conceptualization, funding acquisition, supervision, writing─review & editing.

### Conflict of Interest

The authors declare no conflict of interest.

## Data Availability

The data, tools, and materials (or their sources) that support the protocol are available from the corresponding author upon reasonable request.
